# Molecular Modeling and Molecular Dynamics Simulation of a Packed and Intact Bacterial Microcompartment

**DOI:** 10.1021/acs.jpcb.5c05178

**Published:** 2025-11-05

**Authors:** Saad Raza, Neetu Singh Yadav, Alexander Jussupow, Cheryl A. Kerfeld, Michael Feig, Josh V. Vermaas

**Affiliations:** † MSU-DOE Plant Research Laboratory, College of Natural Science, 3078 Michigan State University, East Lansing Michigan 48824, United States; ‡ Department of Biochemistry and Molecular Biology, College of Natural Science, Michigan State University, East Lansing Michigan 48824, United States; § Environmental Genomics and Systems Biology Division, 1438 Lawrence Berkeley National Laboratory, Berkeley, California 94702, United States; ∥ Biochemistry and Molecular Biology Department, Michigan State University, East Lansing, Michigan 48824, United States; ⊥ Molecular Biophysics and Integrated Bioimaging Division, Lawrence Berkeley National Laboratory, Berkeley, California 94702, United States

## Abstract

Bacterial microcompartments (BMCs) are protein-bound organelles found in some bacteria which encapsulate enzymes for enhanced catalytic activity. These compartments spatially sequester enzymes within semipermeable shell proteins and are packed full of enzyme cargoes and metabolites as they fulfill their function. Coupling together recent SAXS and proteomics work, it is possible to develop molecular models for these microcompartments and interrogate enzyme and metabolite dynamics within. Our primary goal of this study is to quantify the permeability of metabolite glyceraldehyde-3-phosphate (G3P) and dihydroxyacetone phosphate (DHAP) across the BMC shell through classical molecular dynamics simulation. The *Haliangium ochraceum* model of BMC shell (PDB: 6MZX) was used to model an intact BMC of approximately 10 million atoms. Working at this scale presented its own challenges in managing large data sets, with multiple challenges and hardware advances discussed that facilitated this work. Over approximately 750 ns of aggregate simulation, we see multiple permeation events for these metabolites that were added at high concentration through the pores present within BMC shell tiles. When compared to independent permeability estimates for the same metabolites determined through replica exchange umbrella sampling simulations, the permeabilities varied by approximately 3 orders of magnitude. Regardless, the permeability coefficients for both G3P and DHAP are highly similar and very high, such that only very small concentration gradients can be maintained across the BMC shell between the cytosol and BMC interior. The large simulation systems also facilitated comparisons for molecular diffusivity in the crowded environment within the BMC shell. By our estimates, the viscosity within a packed BMC shell is at least 10-fold higher than it would be in neat solution and is the real driver for varying permeability estimates we obtained through simulation. These findings will be used as design inputs for future bioengineering efforts to make products from BMCs, highlighting how permeable BMC shells can be.

## Graphical abstract

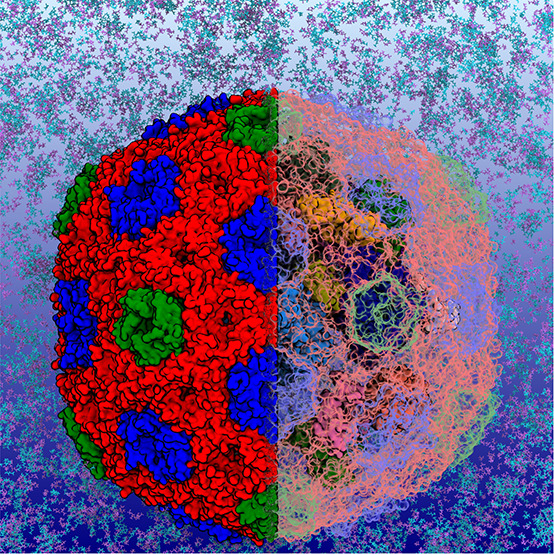

## Introduction

Compartmentalization is essential for all life, allowing gradients to form that can be exploited to store, capture, and convert energy across biology.^1,2^ Although bacteria do not have large membrane-bound organelles like those found in eukaryotes, there are protein shell assemblies called bacterial microcompartments (BMCs) that are believed to compartmentalize metabolism in a wide range of microbial hosts.^3^ Carboxysomes are a type of BMC that encapsulate rubisco and carbonic anhydrase,^4^ facilitating efficient capture CO_2_ by cyanobacteria by reflecting the substrate back toward the encapsulated enzymes.^5^ Metabolosomes are another class of BMCs that encapsulate catabolic pathways^6,7^ such as the use of propanediol^8^ or glycyl radical enzymes.^9^ By sequestering intermediates within the BMC interior, BMCs greatly accelerate catalysis in metabolism^5,10^ and further limit the potential deleterious effects of highly reactive intermediates.^11,12^ Synthetic biology tools are rapidly expanding what these BMCs can do, opening the door to mixing and matching encapsulating BMCs with specific pathways of interest.^13^ Complementary simulation approaches are thus very valuable to answer engineering questions specific to BMC utilization.

A fundamental question around designing BMCs for catalysis are the factors that govern small-molecule permeability for the reactants and products within encapsulated metabolic pathways. BMC shells are composed of hexameric (BMC-H), trimeric (BMC-T), and pentameric (BMC-P) protein building blocks that self-assemble into polyhedral structures.^14^ BMC-T proteins can tiles composed as a monomer (BMC-T^S^) or dimer (BMC-T^D^), with high resolution structures available for many diverse BMC shell proteins.^14–16^ These tiles have central pores that are thought to be selectively permeable to a range of metabolites^17^ or gases.^18^ The central pores of BMC-T^D^ have an arginine salt bridge and an airlock mechanism that can control the passage of large metabolites.^19,20^ These structures were essential to explore selective permeation mechanisms, such as energetic barriers to specific substrates through simulation^21–23^ although observed permeation rates can be quite fast,^24^ even for non-native substrates.^25^

Molecular simulation offers a unique view into these permeation questions for BMCs, as the unparalleled simultaneous spatial and temporal resolution afforded by classical molecular dynamics simulation^26,27^ allows individual molecules to be tracked over time.^5^ Although many of the earliest simulations of BMC proteins were focused on single tiles^21–24^ more recent simulations have followed larger protein subassemblies,^28^ the assembly process itself through multiscale models^29,30^ or detailed studies of tile curvature.^31^ The evolution of BMC simulations parallels the progression of viral capsid simulations, starting from smaller fragments before moving on to full capsid simulations^32,33^ and assembly processes.^34^ The scales between BMC and viral simulations are also similar. Prior all-atom simulations of a full BMC were 2.5 million atoms,^5^ much closer to the 1 million atoms of the satellite tobacco mosaic virus that is a common simulation benchmark^33^ to the 64 million atoms needed to simulate a full HIV capsid.^32^ However, as computing technologies have improved, simulations at this scale have become more common, to the point where they are readily incorporated into primarily experimental studies on the structure and dynamics of BMCs.^24,35^

We will be moving beyond the largely empty shells from prior fully atomic BMC simulations^5,35^ to a packed BMC loaded with proteins and metabolites to learn what new physics and chemistry there is to learn at this simulation frontier. The 11 million atom BMC shell system simulated here is an inferred model from an ensemble of structures fitted to reproduce small-angle X-ray scattering (SAXS) and pair distance distribution data,^35^ which featured adventitiously encapsulated proteins found in *E. coli* within a synthetic BMC shell from *Haliangium ochraceum* (HO).^15,35,36^ Beyond the packed proteins, the model simulated here also include the small molecule metabolites glyceraldehyde-3-phosphate (G3P) and dihydroxyacetone phosphate (DHAP), which are interconverted by triosephosphate isomerase (TPI) as part of glycolysis.^37^ TPI is well characterized and with a high turnover rate, facilitating comparisons between our simulated system and prior experimental studies.^38^ Although TPI itself was not present in the 11 million atom BMC shell simulations, the large simulations with high metabolite concentrations together with complementary Hamiltonian replica exchange umbrella sampling simulations determine permeability for TPI metabolites through the BMC shell protein pores. We further take advantage of this packed system to explore what potential new insight we can gain about metabolite permeability, and metabolite interactions with shell or cargo proteins inside the BMC.

## Methods

Simulating the BMCs required a suitable structure and packaging of cargo proteins inside the BMC shell. Initial coordinates for the protein components are straightforward to obtain from existing cryo-EM structures,^39^ supplemented by structure prediction tools like AlphaFold 2.^40^ Instead, filling this initially hollow shell is the key challenge, both in terms of balancing water across the shell boundary, but also in packing the contents within the BMC shell. These methods will be described in detail, together with a replica exchange umbrella sampling simulation set to determine molecular permeability for DHAP and G3P independently from the large shell simulations described here and run in parallel.

### Shell Preparation and Packing

The initial shell, comprised of BMC-H, BMC-T and BMC-P proteins, was prepared from the 6MZX cryo-EM structure that captured most of the structural details of the HO shell.^39^ However, this structure was missing terminal residues, and so individual chains modeled through AlphaFold2 ^40^ were used to fill out the structure for the shell. This step is identical to the simulations for an empty shell described in prior work,^35^ although the BMC-T^D^ trimers were replaced with BMC-T^S^ in this model, as the BMC-T^S^ were in the construct where TPI activity was observed.^38^ The BMC shell was solvated using TIP3P water molecules in a 466 Å­(*x*) × 466 Å­(*y*) × 466 Å­(*z*) box and the system was neutralized using NaCl and an additional 0.15 (mol/L) of NaCl was also added. Solvation was done using the solvate command and autoionize command for ionization in VMD.^41^ This prototype empty shell is thus ready for simulation, initially with the aim of matching simulation dynamics for this empty shell to SAXS profiles.^35^

This test system highlighted one of the big challenges to working in BMCs. Like lipid vesicles, there is a clear inside and outside of a BMC shell, and so it is possible to add too much or too little water to the inside of the BMC. Unlike lipid vesicles, where water permeation is slow and water imbalances across the membrane can drive vesicle shape changes.^42,43^ BMCs have explicit pores that are highly permeable to water.^5^ Despite this high permeability, we will demonstrate that the BMC shell structure readily deforms due to the osmotic pressure across the shell if the water quantities are not carefully managed. This collapse can be quantified as a net flow of water into or out of the BMC shell, or through internal volume of the BMC shell. One way to solve shell collapse was to selectively add water molecules inside of the BMC shell by a fixed percentage. We do so by selecting the correct number of water molecules to add, and copying those water molecules into the molecular system a second time after applying a small displacement. Replicating the water in this way helped to balance the influx rate observed during test simulations, which lead to balancing of the osmotic pressure for maintaining a stable shell.

Modeling to interpret previous SAXS data suggested adventitious cargo loading inside the BMC shell.^35^ The proteomics data associated with these prior results suggested that the cargo contents were effectively randomly captured cytosolic contents. The *E. coli* cytoplasm is crowded, with approximately 20% of the total internal volume occupied by proteins as in prior modeling approaches.^44^ Thus, for our model, we select the most abundant proteins from the proteomics data set until we arrive at a similar protein density. Concretely, 2 copies of common trimers were included and then the five most abundant proteins were placed relative to their abundance and size from the proteomics data. The smaller peptides were later added to fit and fill the gaps remaining to get the internal occupied volume around 20%. Thus, only 14 of the 26 previously isolated proteins^35^ made their way into the current model, and all were well-structured proteins.

These adventitiously loaded *E. coli* protein cargoes are what we place within our shell models (Table 1 and Figure 1). Structure of the protein cargo were taken from pdb database, otherwise they were built using AlphaFold2.^40^ One peptide oxyR was supplemented by AlphaFold2 for modeling the missing 6 C-terminal residues in the PDB. The *E. coli* proteins were initially placed inside shells via a multiscale modeling protocol using MMTSB Tool set^45^ and CHARMM.^46^ The initial model was further optimized by LongBondEliminator^47^ to eliminate any remaining bad contacts.

**Table 1:** Adventitiously Enclosed Proteins According to the Small Angle X-ray Scattering Profiles and Proteomics Data^35^

Protein name	Uniprot Id	Complex	Protein abbreviation	Copies	PDB-ID/AF2
ATP-dependent RNA helicase DeaD	P0A9P6	Dimer	deaD	1	AF2
Regulator of sigma D	B1XBZ8	Monomer	rsd	2	4XWJ
DNA-binding transcriptional dual regulator	P0ACJ8	Dimer	crp	2	1G6N
HTH-type transcriptional regulator	P0A9F3	Monomer	cysb	1	AF2
3-ketoacyl-CoA thiolase	P21151	Monomer	fadA	3	AF2
d-tagatose-1,6-bisphosphate aldolase subunit	C4ZSH9	Monomer	gatZ	2	AF2
Lactose operon repressor	P03023	Dimer	lacI	3	1EFA
Acyl-UDP-*N*-acetylglucosamine *O*-acyltransferase	B1XD50	Trimer	lpxA	2	1LXA
DNA-binding transcriptional dual regulator	P0ACQ4	Monomer	oxyR	2	AF2/1I6A
Maltodextrin phosphorylase	P00490	Dimer	malP	2	1AHP
FKBP-type peptidyl-prolyl cis–trans isomerase	P0A9K9	Monomer	slyD	3	2KFW
tRNA (guanine-N(7)−)-methyltransferase	P0A8I5	Monomer	trmB	1	3DXX
HTH-type transcriptional regulator	P0ACR4	Monomer	yeiE	1	AF2
Protein yrda	P0A9W9	Trimer	yrdA	2	3TIO

**Figure 1:**
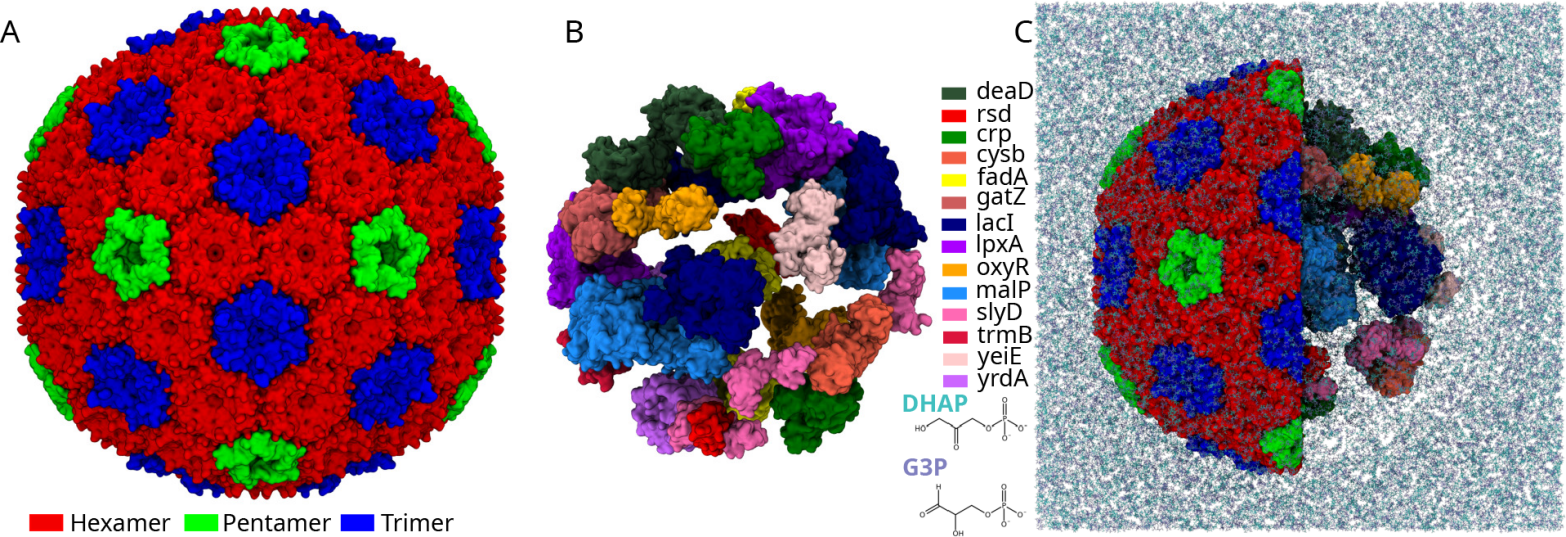
Bacterial microcompartment shell (A) along with the enclosed protein (B) and a representative system for MD simulation setup with 250 mM concentration of metabolites (C). Proteins and metabolites are color coded according to the legend. Proteins are in surface representation and the metabolites in licorice. In panel C the shell is partially shown along with the enclosed proteins and metabolites in the simulation system.

To evaluate small molecule motions, packed BMC shell was also filled with simultaneously with DHAP and G3P with 250 mM concentrations for both in the system, these metabolites were added by replacing the water molecules. These high concentrations facilitate relatively rare transit events to be captured on accessible molecular simulation time scales, as in prior permeation studies for model carboxysomes.^5^ After addition of the small molecules, the VMD solvate plugin was used to add TIP3P water box of size 466 Å­(*x*) × 466 Å­(*y*) × 466 Å­(*z*) box. System was neutralized by NaCl and an additional 150 mM of NaCl was added by autoionize.^41^ Like the empty shell system, we duplicated a percentage of the water molecules using VMD and TopoTools to reduce net water flow.^41,48^

### Equilibrium Simulation Protocol

To gain insight into the permeability mechanics at an atomic level, classical molecular dynamics simulation is utilized to determine what if any metabolites flux can be measured across the bacterial microcompartments. Simulations were carried out using the CHARMM36m force field for the protein components,^49^ coupled to the CHARMM General Force Field for small molecules.^50^ The TIP3 water model was used for the explicit solvent model.^51^ NAMD 3.0b6 with the newer GPU-resident integrator were used as the molecular dynamics engine for minimization and production simulations to maximize performance.^52^ Pressure was maintained using the Langevin piston model at 1 atm.^53^ Temperature was kept constant at 298 K using the Langevin thermostat with 1 ps^–1^ damping. This damping will lower the rate of diffusion for molecules with respect to water dynamics in a different integrator. Hydrogen bonds lengths were constrained with the SETTLE algorithm to enable a 2 fs time step.^54^ Particle mesh Ewald (PME) summation was used to determine long-range electrostatic interactions with a grid spacing of 1.2 Å.^55,56^ Short range nonbonded terms were calculated with a 12 Å cutoff with a switching distance of 10 Å.

Initially every system was minimized using 5000 steps of conjugate gradient in NAMD.^57^ Following minimization, velocities were reinitialized to 298 K and allowed to equilibrate for 1 ns. After initial equilibration, dynamics was run for 250 ns of simulation for each replica.

### System Assembly and Simulation for Permeability Determination through REUS

Beyond the packed BMC shell, the inhomogeneous solubility diffusion model (ISDM)^58,59^ offers an alternative approach to determine molecular permeability. The ISDM’s primary advantage for BMCs is that it can estimate permeabilities from shell fragments alone, shrinking the system size from the multimillion atom simulations needed for equilibrium permeability estimates. Based on prior simulation work to build planar BMC shell models,^28^ we can create a reduced system that features essential BMC symmetry and pore structure by including both hexamer and trimer components of a HO-BMC shell (Figure 2). In our reduced model, there are 3 μ_B_C–H pores and 1 μ_B_C-T^D^ pore along which the DHAP and G3P metabolites can cross the shell surface. By sampling along each pore independently, we can arrive at estimates for the free energy and diffusivity needed to compute diffusion through the ISDM.

**Figure 2:**
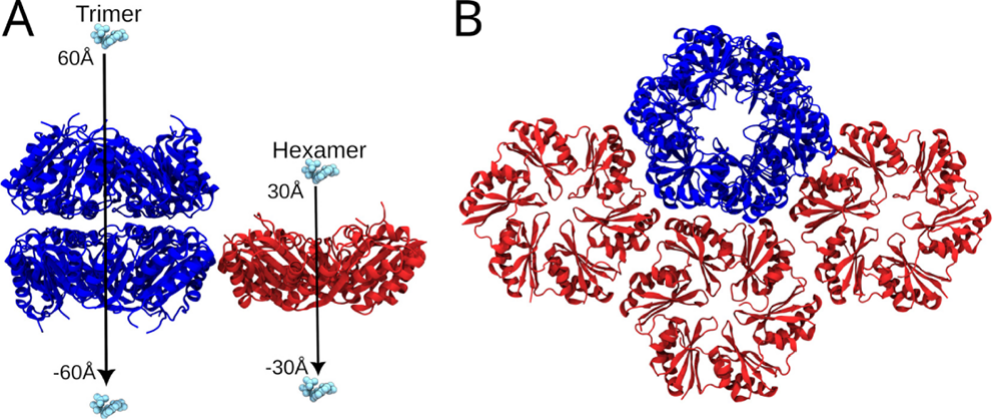
2D system representation of reduced Bacterial microcompartment used for the REUS simulations. (A) The DHAP and G3P molecules were placed at the top of the pores. The collective variable for BMC-T^D^ and BMC-H were set to ±60 Å and ±30 Å, respectively. (B) Top view of the BMC-T^D^ along with the BMC-H.

To make these measurements, 5 copies for a given metabolite were placed in the simulation system, one for each BMC-H pore, and two to sample the upper and lower halves of the BMC-T^D^. The goal is for each metabolite to be harmonically restrained to sample the pore axis, analogous to prior work in carboxysomes.^22,23^ To do so, we first use NAMD 3.0b6 ^52^ to perform steered MD simulations, using the colvars module of NAMD^60^ to drive these metabolites across the pore. Steered MD runs were performed using a spring constant of 10,000,000/kcal/(mol·Å^2^). To eliminate potential protein rocking motions while additional forces are applied, the *z*-positions of protein backbone atoms along a plane were restrained by a harmonic potential with a force constant of 5 kcal/(mol·Å^2^). The small molecules were also restrained from moving >15 Å from the pore center by a cylindrical restraint with a 5 kcal/(mol·Å^2^) force constant.

The SMD simulations were performed in two steps. In the first step, metabolites that eventually transit through the hexamers were held at −30 Å, while the trimer side metabolites were pulled from solvent to the center (60 to 0 Å), while the other was held at −60 Å. In second step, the metabolites were pulled through the hexamer pore from −30 to 30 Å, while the two metabolites being pulled through the trimer pore were moved from −60 to 0 Å, 0 to 60 Å, respectively. The result of the second step is used to seed the initial coordinates for the REUS simulations that are used to determine the free energy and diffusivity profiles. To prevent the metabolites from leaving their assigned pore, an additional cylindrical restraint was applied as an additional distance XY collective variable to keep the metabolites within 15 Å of the pore center with a 5 kcal/mol/Å^2^ force constant.

For REUS simulation both DHAP and G3P molecules were restrained with a 5 kcal/mol/Å^2^ spring constant in 128 equally spaced umbrellas along the *z* axis going through the central pore of shell fragments. The reaction coordinates spanned from 60 Å to −60 Å for the trimer dimer, split across two different umbrella sampling sections, and 30 Å to −30 Å for hexamers. In this sampling scheme, the windows are approximately 0.5 Å wide, which has worked well in prior simulations to calculate permeability across lipid bilayers.^61,62^ During REUS sampling, exchanges between adjacent replicas were attempted every picosecond on alternating pairs of replicas, for an effective exchange attempt every 2 ps (500 ns^–1^). This high exchange rate is in line with best practices for replica exchange simulations.^63^

### Analysis

The data from molecular dynamics simulation was visualized and analyzed using python enabled VMD 1.9.4a58.^41^ Python enabled VMD provides access to the numerical libraries like numpy or scipy^64,65^ graph analysis libraries like NetworkX,^66^ and plotting tools like matplotlib.^67^ These tools and libraries are used together to analyze both the equilibrium and nonequilibrium simulations.

From the equilibrium simulations, we can determine multiple quantities of interest. To assess structural stability, we go beyond standard RMSD metrics to track the interior volume of the BMC shell using the net change in the number of water molecules inside and outside the shell. In VMD, this can be measured by using geometric criteria to evaluate the number of water molecules that are enclosed within the shell, and tracking that quantity over time. We also track the interior volume, computed using the measure volinterior command.^68^

Shell–Shell and Cargo–Shell interaction are tracked over time using a differentiable coordination number definition (eq 1), originally used to quantify native contacts^69^ but since applied to quantify close contacts in a number of biological systems.^28,62,70–74^

1 $$C=\overset{n}{\underset{i=1,j!=i}{\sum }}\frac{1}{1+e^{5\left(d_{ij}-4Å\right)}}$$

Equation 1 operates on atom pairs between distinct atoms *i* and *j*, summing the contacts based on the distance between *n* heavy atoms between molecules of interest. This provides insight into the time evolution of the packed BMC shell, particularly novel interactions that may not exist in the initially built state.

Interaction networks between cargo proteins were determined by framewise analysis for the individual trajectories. Two cargo proteins were judged to be in contact if any of the alpha carbons in one protein were within 10 Å of alpha carbons of the other protein. This analysis is efficiently calculated by VMD^41^ using the measure contact command. To better visualize these interactions, we build graph representations built in NetworkX^66^ and use Gephi^75^ version 0.10.0 for visualization. Proteins are arranged as the nodes on an undirected graph, arranged in Gephi^75^ using the ForceAtlas2 ^76^ algorithm. The edges weight influence was set at 0.01 and were assigned a scaling of 10 for repulsion with no overlaps.

From an analogous measurement, we also calculate the nearest neighbor as well. Nearest neighbors for a cargo protein were calculated by getting the euclidean distance between peptides in different multimers or monomers between any atom pair in the protein. Distances were sorted in ascending order and average value observed for the first 10 neighbors were chosen for visualization, including as a histogram.

Diffusion is another quantity of interest, particularly as the solute concentrations inside and outside the shell are very different. Since our starting geometry is unlikely to be perfectly equilibrated initially, we estimate the diffusion coefficients at specific instants in time, rather than fitting a line to the mean squared displacement. To measure the instantaneous diffusion, we measure the mean squared displacement (MSD) for water, DHAP, and G3P over discrete intervals and estimate the diffusion coefficient for a specific time interval (Δ*t*) as follows:

2 $$D\left(\Delta t\right)=\frac{\mathrm{MSD}\left(\Delta t\right)}{6\Delta t}$$

This is just a reformulation of the typical Einstein diffusion relation in three dimensions.^77^ We can vary the windows from 0.5 to 10 ns in length, to evaluate if there are strong diffusive changes over short versus longer time scales. This approach allows us to explore when the molecular simulation system is fully equilibrated, and track changes in diffusion coefficients over time. In addition, as we know whether the particles are initially inside or outside the BMC shell, we can compute different estimates for D from the subpopulation of molecules inside or outside the BMC shell.

There are a few caveats to our diffusion analysis. We do not explicitly correct for PBC box size in our simulations, although these size effects drop off as the simulation box grows.^78^ TIP3P water also diffuses faster compared to water in reality, and has been reported to diffuse 2.4 times faster.^79^ Faster diffusion of water also accelerate the diffusion of protein and metabolites in the system by a similar amount.^80^ While we could adjust our diffusions down by a factor of 2.4, we report the diffusion directly from the trajectory, and depend on the reader to know that the diffusion we report is too high to be realistic.

#### Permeability Calculations

While most of the analysis is derived strictly from the geometry of the molecular simulation system over time, the parallel equilibrium and nonequilibrium simulations offer two independent pathways to estimate small molecule permeability through these molecular simulation systems. In this context, the permeability coefficient *P* is a factor that relates the molecular flux *J* (shown here in units of molecules/time) to the product of the surface area *A* between two compartments and the concentration difference Δ*C* between the two compartments.

3 J=PAΔC

Elsewhere in the literature, *J* is written as a flux per unit area,^81^ but we think it is important to explicitly think about the size of the surface between the inside and the outside of the BMC, and so explicitly include the area term within eq 3.

Counting transitions between the inside and outside of the shell directly translates into permeability, as the transition rate *r* and solute concentration in water *c* are related to the permeability *P* as follows:^81^

4 $$P=\frac{r}{2c}$$

It is important to emphasize here that the number of transitions is anticipated to linearly grow with the surface area of the BMC, and so the transition rate *r* in eq 4 is normalized by the total surface area *A* for the BMC. Thus, if there are *x* transitions over *t* time, eq 4 expands to

5 $$P=\frac{x}{2cAt}$$

In practice, the concentration *c* is simplest to think of in terms of molecules per Å^3^, as the surface area computed from VMD will often be in units of Å^2^, and the number of transitions translates directly into a number of molecules.

The equivalent expression for permeability *P* from the ISDM is based on the free energy *G* and diffusivity *D* computed along a specific reaction coordinate.^59,82^

6 $$P={[\int_{\xi_{l}}^{\xi_{u}}\frac{\exp\left(\Delta G\left(\xi \right)\beta \right)}{D\left(\xi \right)}d\xi ]}^{-1}$$

We estimate the free energy profile to satisfy eq 6 via BayesWHAM.^83^ The local diffusivity is estimated from the variance and time autocorrelation along the reaction coordinate.^84^ An exponential fit to the autocorrelation function at time scales smaller than the exchange frequency is used to calculate the temporal autocorrelation in order to account for the exchanges introduced by REUS sampling. This has been used successfully for membrane permeation studies^85,86^ which produced results that were generally in line with small-molecule diffusivities for low molecular weight hydrocarbons that were measured experimentally in an aqueous environment. With diffusivity and free energy in hand, we use eq 6 to numerically integrate the respective profiles to obtain a permeability estimate.

It should be emphasized that the direct integral from eq 6 is not immediately comparable to the permeability determined via counting. Whereas metabolites in equilibrium simulations are unrestrained, the REUS simulations feature an additional cylindrical bias described above to keep the small molecules lined up with the pore. The confinement to a cylinder means that the permeability is larger than it otherwise would be, since an unrestrained molecule would explore parts of the protein surface that are impassible. Past simulations in carboxysomes have demonstrated that only the smallest molecules can traverse the interface between proteins, and larger molecules must traverse the pore.^5^ If we therefore assume that the small molecules would be fully impermeable outside of the pore, the effective permeability (*P*_eff_) is the computed permeability along the pore multiplied by the fraction of the total tile surface area that the confining cylinder with radius *r* occupies. For our systems, *P*_eff_ is given by

7 $$P_{\mathrm{eff}}=\left(3P_{H}+P_{T}\right)\frac{\pi r^{2}}{A}$$

In this case, *A* is the total surface area for the BMC shell surface, which is the product of the *X* and *Y* dimensions for our simulation box, which covers all 4 pores present in the BMC shell fragment under simulation. Since there are four pores present in our simulation system (one BMC-T pore and three BMC-H pores), there are four confining cylinder areas to consider, however since they are all the same size, the key scaling factor is the $\frac{\pi r^{2}}{A}$ . In our simulations, *r* is 15 Å and the surface area for the BMC sheet is approximately 16166 Å^2^, yielding a scaling factor of 0.0437. Once we account for the four pores in the molecular simulation system, the permeability through the combined surface is only about 16% of what we compute through the cylindrical restraint.

### Performance Comparison

We feel at this juncture it is important to give a sense of the performance for these simulations with reasonably modern hardware, and to be transparent with how much computational effort is behind the simulations we present here. The recent implementation of the GPU resident integrator in NAMD3 ^52^ has been a substantial advance in the efficiency of large scale simulations, as even a single GPU is now able to simulate a few nanoseconds a day for the ∼10 million atom system considered here (Figure 3). Notably, for large systems like this, NAMD scales effectively linearly. Comparing the RTX3090 and A100, which are both Ampere GPUs, their performance is basically identical when adjusting for the number of GPUs present, as a hypothetical workstation with 4 RTX3090s would expect 4.68 ns/day of performance. Notably, differences in CPU layout can show up occasionally, as the Delta system at NCSA and the Perlmutter system at NERSC share the same AMD EPYC 7763 base, but Delta has two of them, while Perlmutter only has one. As a consequence, you occasionally see small performance reductions on Delta when data needs to travel between CPUs in order to communicate between the GPUs.

**Figure 3:**
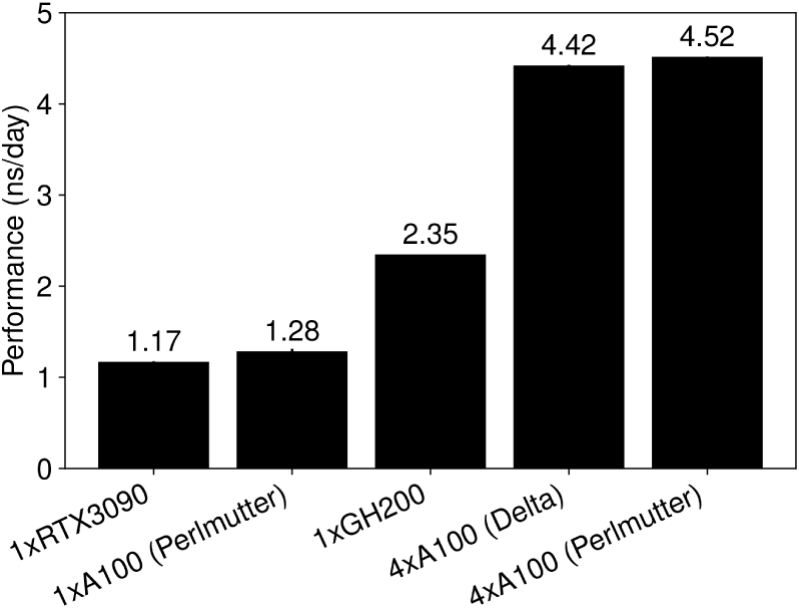
Benchmarks for 10 million atom BMC simulations using NAMD 3 on varying NVIDIA hardware, either consumer/gaming graphics cards (RTX3090) or server/enterprise cards like you would find on supercomputing centers (A100, GH200).

The level of performance observed in Figure 3 is roughly equivalent to the GPU-accelerated performance for a 100k atom system in the early 2010s, and means that 100 ns of sampling is roughly 1–2 months worth of computing effort on just a single server. By contrast, each replica within the REUS simulations (a 220k atom system) runs at approximately 45 ns/day on a single GPU despite the considerable overhead of moving data between CPU and GPU to facilitate collective variables calculations. Thus, each REUS simulation will finish within a day or two given enough available computing time, although queuing policies usually make this take longer.

Another consideration is power efficiency. If what we really care about is a defensible estimate for the permeability, the equilibrium simulations for the 10 million atom systems will consume approximately 2–5 GJ (650–1400 kW h) of energy depending on the length of the simulation needed to observe multiple transition events, with a single GH200 GPU that we use for these simulations consuming approximately 900 W under load. We need 128 A100 GPUs for about a day to estimate permeability through REUS. Since each GPU consumes 400 W under load, that works out to be about 4 GJ (around 1200 kW h), or about the same energy cost if we neglect the power going to the CPU and other peripherals such as networking or storage. While prevailing electricity rates are highly dependent on location, the 12.68 cent per kW h average across the US^87^ means that the electricity cost of determining permeability by simulation is under $200 per molecule.

## Results and Discussion

### Shell Stability *In Silico*

Water plays a crucial role in cell structure by enforcing hydrophobic behavior and building up osmotic pressure.^88,89^ Water is permeable through the pores of a BMC shell in simulation,^5^ however as we learned from simulations of an empty BMC shell, the shell will deform due to osmotic pressure differences unless the water is balanced inside and outside the shell.^35^ What worked when the HO shell had no cargo was 4% extra water molecules were added inside the BMC, as that was enough to negate the tendency over long time scales for water to flow into the shell under default conditions (Figure S1).

However, once cargo was added, even more water molecules needed to be added to the inside of the BMC shell to maintain the structural integrity of the shell. Short test simulations yielded strong structural deformations when only 4% or 10% of the interior waters were replicated, or if too many water molecules are replicated (Figure 4). There is a equilibrium condition where the osmotic pressure is approximately balanced between the two sides of the BMC shell, and the shell itself is stable. In our experience, the easiest way to establish this condition is simply through trial and error, running through multiple conditions until the net inbound water flux is basically zero over the first few nanoseconds of simulation (Figure 5). Particularly for cargo-loaded BMCs, or for viruses that are similarly packed full, there is not currently a simple way to check if structures are correctly solvated.

**Figure 4:**
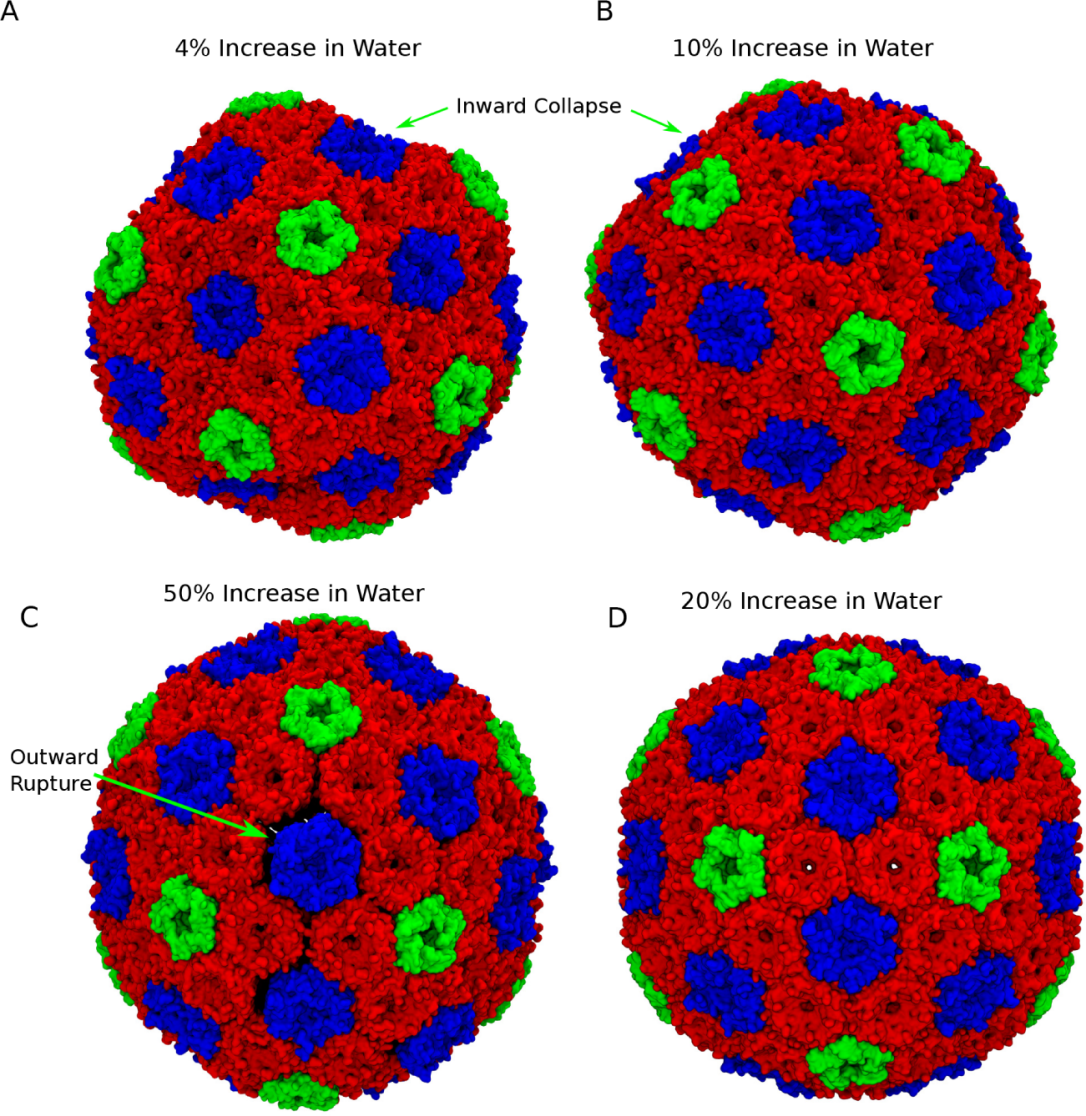
Visual impact of internal water concentration on structural stability. In all cases, we replicate a fraction of the water molecules within the protein shell to try and balance the osmotic pressure. When not enough water is present, as in the 4% or 10% cases in subpanels A and B, the shell buckles inward due to the imbalanced osmotic pressures on either side of the shell. If too much water is present, such as when we replicate every other water molecule (50%, C), the excess water can rupture the shell. Only when the osmotic pressure difference is small do we have a stable simulation, such as the 20% replication conditions in panel D. In all panels, the protein shell is colored according to identity, with red for the BMC-H units, green for the BMC-P units, and blue for the BMC-T units.

**Figure 5:**
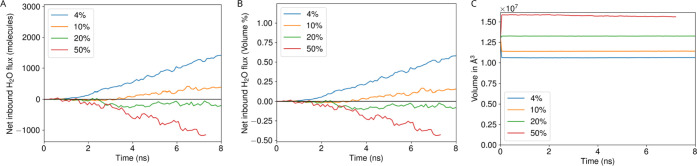
Water ingress rate for different concentration inside BMC, reported either based on the number of water molecules (A), or equivalently as a percentage of the total number of waters within the BMC interior (B). Internal volume of the BMC shell over time for different concentration (C).

The reasons for this issue are numerous, and come from the mismatch in constraints between typical simulations and the large simulations we run here. Most protein solvation schemes are meant for the use case where the protein is isolated in a water box or a membrane. In those cases, the exact water placement near the protein surface is not critical, since water will readily reach those spaces after only a short simulation. Thus, solvation methods are possibly too conservative in placing water near the protein where it might distort the protein structure. As a consequence, empty pockets will appear within the BMCs after only a short simulation if the default behavior is left intact. Fixing protein positions and trying to let the water equilibrate would not be ideal, as the number of waters that need to be transported to equalize the pressure would be thousands of water molecules (Figure 5A), and the rate at which waters are forced through the pores will not make up that difference in a few nanoseconds.

Instead, by just adding more waters in a ″guess and check″ approach leverages fast water rearrangement within a compartment, effectively expanding it by adding more atoms in the interior. These volume changes are very fast (Figure 5C)! Similar approaches have been developed for both atomistic^90^ and coarse grained membrane vesicles,^43^ where water molecules were similarly added until the structure stopped changing shape at short time scales. We suspect that this resolvation step could be automated, possibly by using measure volinterior from VMD^68^ to track volume changes. However, given the resource demands for running these large systems, keeping a human in the loop to look for structural deformations appears to be prudent at this juncture. Regardless, the remaining full shell simulations were run from the stable structure, where 20% of the interior water molecules were replicated to balance the osmotic pressure across the shell.

### Permeability

BMC shells have the potential to accelerate catalysis by kinetically trapping intermediate metabolites within the shell interior, while being permeable to reactants and products. Permeability coefficients for the small molecule metabolites involved in catalysis are thus essential engineering inputs to designing BMC shells that maximize the investment by the cell in these large protein cages. While incredibly difficult to measure through experiment, simulation offers two paths to determine these essential parameters. The first uses the inhomogeneous solubility diffusion model (eq 6), using free energy and diffusivity determined from biased simulation as the input. The second counts explicit crossing events observed in simulation to explicitly determine the permeability coefficient (eq 5). Here, we use both methods to estimate the permeability coefficients for the DHAP and G3P metabolites that are interconverted by *tpi* within glycolysis.^37^ Note that while nature does encapsulate glycolysis within a BMC, these metabolites are reasonable proxies for small organic molecules metabolized within some BMCs.

#### ISDM Permeability Estimate

From our REUS simulations, we can estimate both the free energy and diffusivity needed to estimate permeability through eq 6. Since we have both hexameric and trimeric pores within our initial system (Figure 2), and these pores were sampled independently, we can arrive at specific estimates for the permeability through each subunit within the BMC. Both DHAP and G3P are very similar, carrying the same charge, molecular weight, even the same number and type of atoms, with only a small rearrangement in bonding facilitated by *tpi*. Thus, we should expect the free energy and diffusivity profiles to be similar between these two molecules, with perhaps small changes with how the environment interacts with the ketone or aldehyde groups found on DHAP and G3P, respectively. This is indeed what we observe in both the free energy and diffusivity profiles, which are largely comparable between both molecules (Figure 6).

**Figure 6:**
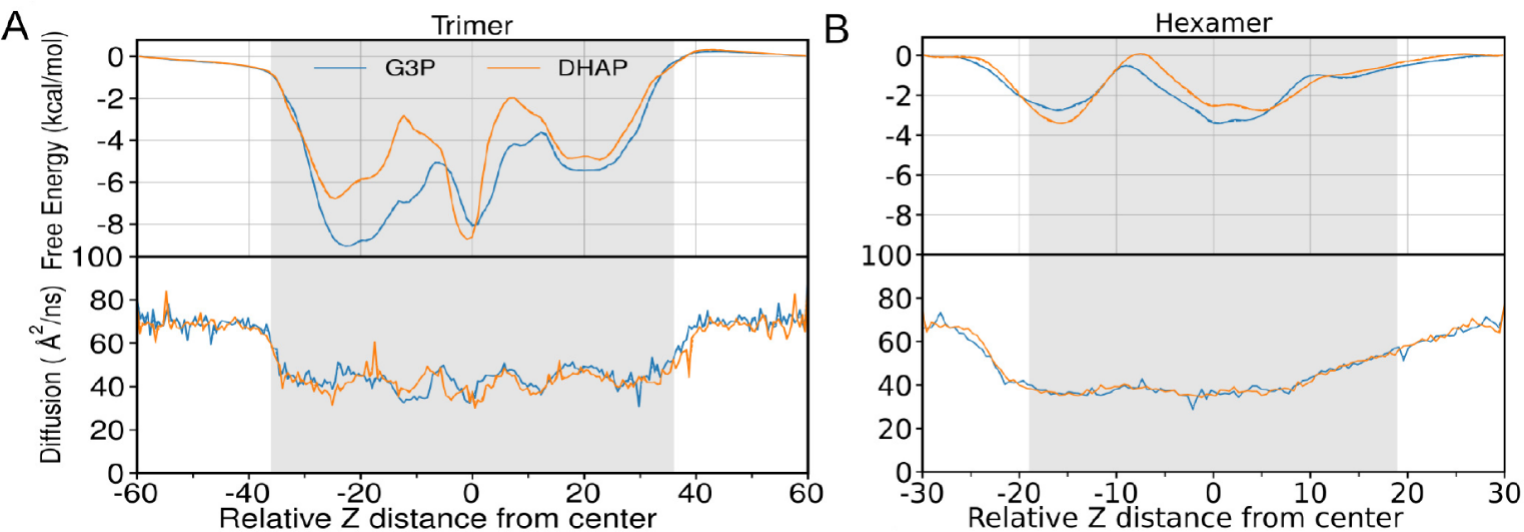
Free energy and diffusion plot for G3P and DHAP molecules crossing the (A) hexamer and (B) BMC-T^D^ pores. The curves are obtained from the REUS simulation after removing initial 20 ns of data collection as an equilibration period. Total simulation time for DHAP and G3P is 80 ns. The shaded gray area is the region where the BMC shell protein will be present, with negative *Z* values indicating the inside of the shell and positive *Z* values on the outside of the shell. Standard errors for the free energy profile from REUS are drawn as semitransparent regions, which are rarely visible outside of the main line. The dashed line indicates the spline fit used to numerically integrate eq 6 to tabulate permeability coefficients.

The hexamer profiles are where we have the most data, as the simulation system had three independent copies where permeating molecules simultaneously sampled the permeation reaction coordinate (Figure 2). The free energy profiles are within 1 kcal/mol of one another across the full range, with the diffusivity profiles effectively superimposed on top of one another (Figure 6). Notably, the region with the highest free energy is in solution, indicating that DHAP and G3P interact preferentially with the protein. As both DHAP and G3P carry negative charges, and the HO shell we are working with has positively charged patches thought to interact with negatively charged substrates,^39^ this is a reasonable result, even though it is surprising that these minima appear both on the ″inside″ (−*z*) and ″outside″ (+*z*) sides of the hexamer bottleneck at around −8 where the pore is narrowest.^28^ Interpreting the free energy, the likeliest location for DHAP and G3P are on the surface of the protein, where diffusion is approximately half of what it would be in solution.

The trimer profiles are noisier, largely due to lower sampling compared to the hexamers. While the +z and −*z* halves of the reaction coordinate are sampled independently, analogous to a common trick for lipid bilayer permeation calculations^72,91^ there is still only one BMC-T^D^ present in the simulation. This limited sampling may be the origin of the larger differences between G3P and DHAP free energy profiles (Figure 6), although real quantitative differences cannot be ruled out. Importantly, three well-defined minima is exactly what we would expect as we pass through the BMC-T^D^, as there are two narrow bottlenecks separating three larger compartments in this double stacked trimer.^28^ The protein residues found at the energy minima are represented in Figure S34. The interaction strength between the small molecule and the trimer are much larger on average, as the free energy profile go below −8 kcal/mol at their minima. Similar to what happened for the hexamers, the trimer diffusivity profiles range from approximately 70 Å^2^/ns in solution to a little under 40 Å^2^/ns within the interior of the protein where movement is impeded by the presence of the protein.

All told, once we integrate the profiles from Figure 6 using the ISDM formalism from eq 6, the permeability coefficients are estimated to be in the single digits when expressed in cm/s (Table 2). Such a high permeability coefficient is comparable to the permeability coefficients for lipophilic small molecules across a lipid bilayer^61,62,72^ and would suggest that the built-up concentration gradients between the inside and outside of the BMC must be quite small.

**Table 2:** Permeability Comparison Table for Both the Transition Counting Method from Explicit Simulation (Eq 5) and the ISDM Method from Eq 6 ^a^

	Explicit simulation		ISDM estimate		
Molecule	Transitions	*P* (cm/s)	*P*_H_ (cm/s)	*P*_T_ (cm/s)	*P*_eff_ (cm/s)
DHAP	15	0.0055 ± 0.000	23.9 ± 0.2	13.5 ± 0.1	3.7 ± 0.2
G3P	15	0.0055 ± 0.002	33.7 ± 0.2	13.3 ± 0.2	5.0 ± 0.3

a To better compare between the permeability estimates, we need to adjust for the effect of the cylindrical confinement on the ISDM method, following the procedure from eq 7 to determine an effective permeability *P*_eff_.

### Permeability Estimate from Direct Simulation

From our large BMC shell simulations with high concentrations of both DHAP and G3P, we can independently estimate permeability by tracking individual permeation events observed within our trajectories, such as the example in Figure 7. By tracking the radial position of individual metabolites compared with the simulated BMC shell, we can readily identify transition events within our trajectories, which correspond to where the small molecule transits the pore (Figure 7). Across the 750 ns of aggregate simulation time for the intact HO shell from three 250 ns replicates each, there are 15 crossing events for G3P, and 15 for DHAP (Table 2). Between individual replicates, the number of crossing events varies between simulation replicas. For DHAP, 5, 5, and 5 crossing events were observed across the three simulation replicates, while the G3P simulations showed 2, 5, and 8 crossing events (Figures S2–S31). This is normal variation due to sampling, and indicates that our estimated permeability coefficient via eq 5 is reasonably accurate from just three replicates.

**Figure 7:**
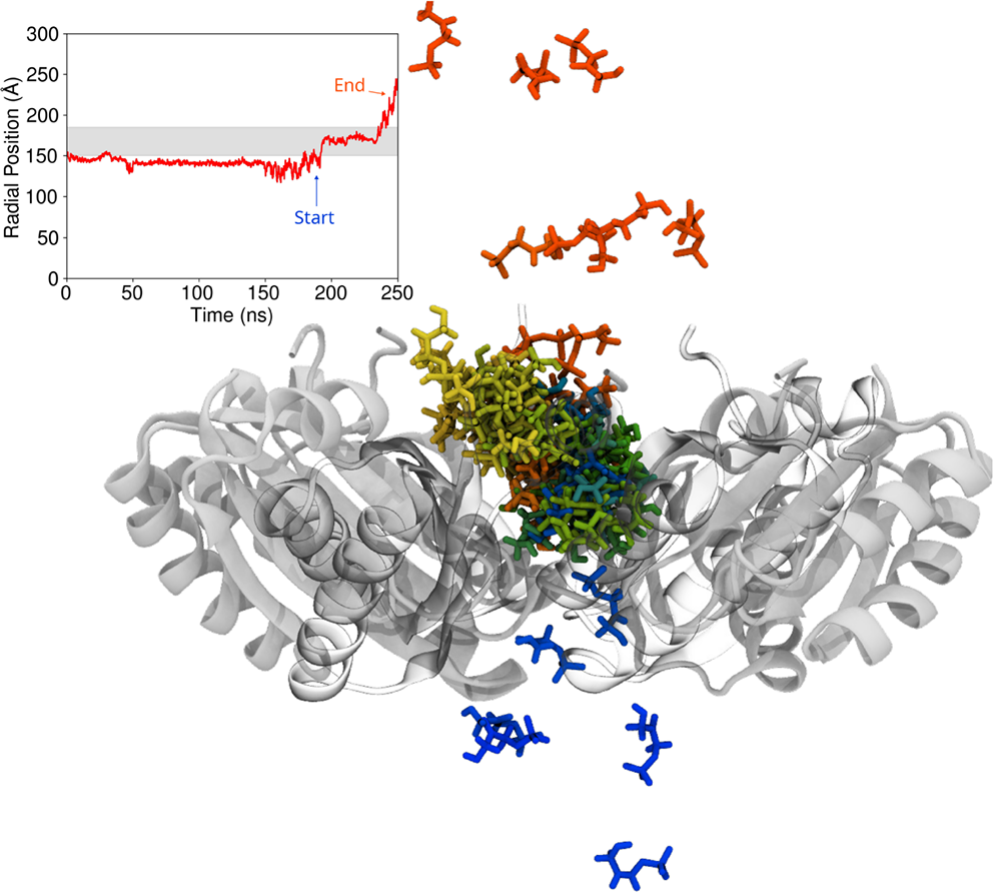
Example of a transit event through the pore of a hexamer, in this case DHAP through a hexamer. The full timeseries for the radial position is shown in the upper right, but we focus on the region toward the end of the simulation where this molecule transits through the pore. The molecular image focuses on the single DHAP molecule that corresponds to this timeseries, drawing this molecule multiple times in various colors and orientations as it transits through the hexamer pore. Bluer colors are nearer to the start of the trajectory snippet where the transition takes place, while redder colors are nearer to the end of the trajectory snippet. All transition traces are shown in Figures S2–S31, including the trace shown here, which is Figure S12.

The biggest surprise from Table 2 is the difference in magnitude between our independent estimates for the permeability coefficients. The estimates obtained by counting permeation events are nearly 3 orders of magnitude slower than the permeability estimates obtained via the enhanced sampling simulations. While it is well-known that ISDM estimates can overestimate the true permeability coefficient^91,92^ particularly when compared with explicit counting methods,^93^ 3 orders of magnitude demand a more thorough investigation to compare and contrast the different assumptions baked into these simulations.

The obvious difference here is the concentration of the metabolites, particularly when we are explicitly tracking crossing events. In these simulations, the 250 mM concentrations for DHAP and G3P are far higher than would occur in a natural system, with estimates from the literature typically in the 1–400 μM range.^94–97^ The high concentration during the equilibrium simulations for the intact shell is in stark contrast to the 5 total DHAP/G3P molecules present during the REUS simulations, which works out to be approximately 5 mM based on the 60,000 water molecules present in the simulation system. A more concentrated system would have slow diffusion, which would reduce the permeability.

An additional wrinkle unique to BMCs is that the high concentration for metabolite molecules may lead to queuing delays, as the small molecules physically cannot pass one another in the limited number of pores in the HO shell. At the high metabolite concentration we use here, nearly all of the places where a metabolite could plausibly be are filled. Indeed, if you follow the radial distribution of metabolites, there are distinct peaks at the entrance and exit for the shell as the metabolites ″wait their turn″ for access to the shell interior (Figure 11B). Thus, it stands to reason that the rate at which metabolites might cross the shell might not be linearly related to the small molecule concentration as anticipated by eq 5, as transport at high concentration may saturate the pore and depend on multiple small molecules to coordinate and allow a transit event. However, if we reduce the concentration in our simulation by the factor of 1000× to bring it in line with real concentrations, we would need far more simulation time to observe a permeation event, which is not currently feasible.

Another point of interest is whether the free energy profiles sampled at equilibrium are even analogous to the free energy profiles sampled via REUS. Now, since we have different trimer structures in the equilibrium (a BMC-T^*S*^ T1) and REUS (a BMC-T^*D*^ T2/T3 stack), we can only really compare the free energies along the hexameric pore (Figure 8). The profiles in Figure 8 are largely similar, with peaks and valleys near the same location with comparable magnitudes. There are some subtler changes, such as the location of the peak changing by approximately 5 Å along the hexamer normal axis. If we take these results at face value, the values from the equilibrium simulation are about 1.5–2 kcal/mol higher more or less across the board, which would decrease the permeability by about a factor of 50 in the inhomogeneous solubility diffusion model. If we evaluate the diffusion along the pore from equilibrium (Figure S32), and compare against the diffusivity profile in Figure 6, we see that the profiles differ with REUS computing 4× faster diffusion.

**Figure 8:**
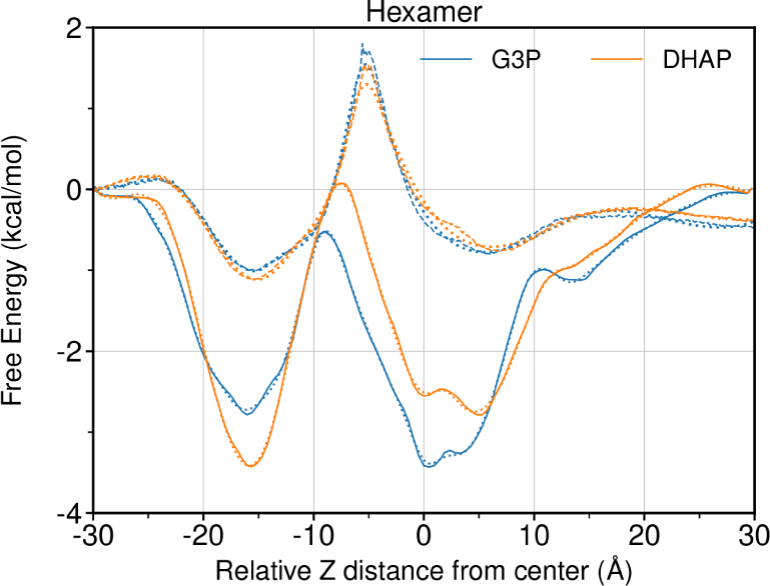
Comparison of free energies obtained from analyzing the equilibrium trajectory (dashed lines) and REUS trajectories (solid lines) of the hexamer pore free energy. The color for the line indicates the metabolite, while the line style indicates which trajectory the free energy is from. The dashed lines are used to represent the equilibrium trajectory free energies determined through Boltzmann inversion of the probability distribution for metabolites within the 15 Å cylinder equivalent to what was sampled in REUS. The solid line represent the free energy estimate from the REUS sampling. Standard errors for the free energy profile are drawn as semitransparent regions, which are rarely visible outside of the main line for REUS data. Dotted line is indicating the spline fit used to numerically integrate eq 6 to tabulate permeability coefficients. Negative *Z* values represent the inside of the BMC shell and positive value outside the shell.

Both of these errors are in the same direction, leading to REUS overestimating permeability, but only by a factor of 200 or so, not the 1000× seen in Table 2. This brings us back to our earlier rationale, where molecules under high concentration queuing for access to the pore physically block the pore and reduce permeability. Combined with the slightly different measurements between REUS and the equilibrium trajectories, that might be enough to shift the position of the peaks and valleys somewhat. For the equilibrium trajectories, the *z*-positions for all atoms are binned independently to contribute to the probability distribution that leads to the free energy profile. Thus, a molecule that is curled up right below or right above the bottleneck would contribute very little probability density to the bottleneck itself. Conversely, the REUS trajectories are biased based on the molecular center of mass, and so similar poses that span across the bottleneck would be seen to sample the midpoint, and so these small subtleties in what is actually measured may contribute to a difference in the free energy profile.

However, it may not be reasonable to make these comparisons in the first place, as free energy depends on concentration. The concentrations we have in equilibrium and REUS simulations are very different, ranging from a few mM to 500 mM in aggregate. An interesting follow-on experiment would be to measure permeation events in a small sheet structure, analogous to what we are using for the REUS calculation. However, as there would be fewer pores than in the current model, we would expect fewer transition events at similar concentrations, which would require much longer simulations to remedy.

As shown in Figure 8, the free energy profiles of the metabolites exhibit elevated free energies near a specific bottleneck within the pore, with pronounced minima located at approximately 5, −15 Å on either side of this bottleneck. These minima are caused by positively charged amino acids in the BMC pore, visualized in Figure S33 with the contacts quantified in Figure S34. The minima are also recovered in the 2D PMF maps for DHAP and G3P (Figure 9), where the global minima—indicated by blue regions—are consistently positioned near 5, −15 Å. There is a clear difference in the overall free energy on either side of the shell, with the interior having a lower free energy (bluer color) than the exterior. As both DHAP and G3P are negatively charged, these substrates may be responding to the local electrostatic potential difference between the pore interior and exterior (Figure S35), drawing the negatively charged substrates to the inside of the pore spontaneously. Notably, the relatively low free energy barrier separating the inside and the outside of the shell through the pore regions suggests that the preferred translocation pathway for the metabolites involves crossing through the pore, rather than any potential motion through the interface of the protein components as in prior work on gases.^5^

**Figure 9:**
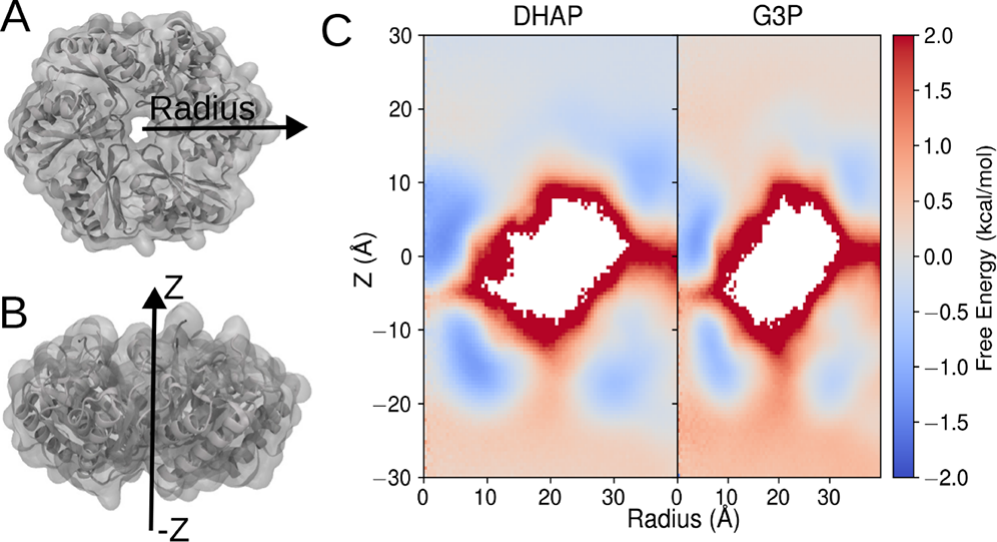
2D free energy surfaces for molecular transport across the hexamer in BMC. Panels A and B show the two dimensions (radial distance away from the pore center, and pore normal axis (*z*)) along which we bin observations to generate the free energy profile. Free energy is generated by using *G*(*z*) = −*k*_B_ *T* ln­(*p*(*z*)). The probabilities have been radially reweighed to account for larger surface area at larger radii. We intentionally chose a diverging colormap centered at zero where the minimum free energy sets the range of possible values. Thus, some deep red points have free energies that exceed 2 kcal/mol.The hexamer pore region extends from from −8 to +8 Å along the *Z* dimension (negative *Z* would be inside the shell, positive *Z* would be outside), and 0 to +8 Å along the radial direction. The white areas where the substrates are never present are where the protein is found.

While the difference in the estimated permeability coefficients may be larger than ideal, we can conclusively say that the permeability coefficient is quite high. This finding adds to the existing literature highlighting rapid transit across BMC shells, even for non-native molecules.^5,25^ While there are some instances in the literature where BMC modifications lead to growth defects attributed to changes in permeability,^98^ the concentration differences that can be supported by permeability coefficients between 10^–2^ and 1 cm/s are very small. If we assume that enzymes within the shell consume 1 molecule per second, using eq 3 we can work out that an approximately spherical shell with a 19 nm radius would have a steady state concentration gradient of approximately 70 nM across the HO shell. This is a really small concentration gradient, and suggests that catalysis within the shell is unlikely to be limited by permeability considerations. Prior *in vitro* studies have already demonstrated DHAP to G3P interconversion within a HO shell,^38^ and so the high permeabilities determined through simulation are quite reasonable.

### Correlated Motions and Diffusion within BMCs

One of the exciting things offered by simulating the complete HO BMC at atomic resolution is tracking motions and interactions at the atomic scale. While this study is not the first to quantify how diffusion slows with greater crowding,^99,100^ we do have a very stark contrast between molecular motions inside and outside the BMC shell (Figure 10). We first employed the standard method to calculate diffusion, using the first frame as reference tracking the mean squared displacement. Displacement outside the shell follows a linear thread that is indicative of normal diffusion, but the mean squared displacement cannot grow for molecules trapped within the BMC shell as the simulation is extended (Figure S36). Instead of measuring the MSD from a fixed reference point, we are measuring the MSD over increasing time scales, from which we can readily check for anomalous diffusion. From Figure 10A, we see that the MSD grows basically linearly with time so that the ratio between MSD and a specific lag time quickly converges. As we increase the time scale for the lag times, the displacement start to reduce faster for water inside the BMC shell, as metabolites are largely but not perfectly confined to remain inside the shell. By sweeping over multiple lag times, we find that the 2.0 ns lag time is a reasonable time scale which can capture anomalous diffusion and low variation in the MSD observed for each replica. Thus, our diffusion estimates in Figure 10B use these 2 ns lag times, and show that the crowded environment inside the BMC has a diffusion coefficient that is about 20% smaller than what is found outside the BMC. This reduced diffusion for small molecules is a smaller effect than is possible, as estimates for protein diffusion at 20% occupied volume fraction can cut diffusion by 60–75%.^101,102^

**Figure 10:**
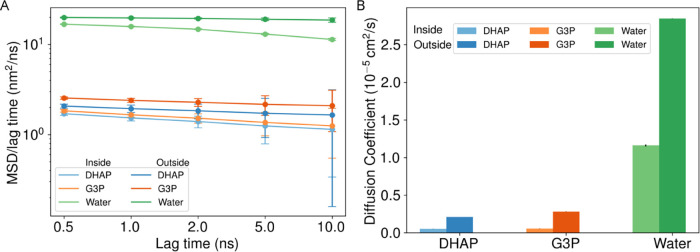
Mean Squared Displacement over different lag time (A) and (B) Diffusion coefficient calculated from eq 2 for DHAP (Blue), G3P (red), and water (Green). Darker colors represent the MSD or diffusion coefficient outside the BMC shell and lighter colors represent diffusion inside the shell. Standard error has been shown in the bar plot of the diffusion coefficient.

What we are missing from the discussion above is what happens when a metabolite encounter the BMC shell surface. Past experiments have suggested that diffusion is further modified at the interface with lipid bilayers,^103^ but we simply do not have enough sampling for the proteins to be able to make that kind of comparison. Instead, follow-on simulations at a coarser resolution would be ideal to quantify the impact of the shell itself on protein diffusion. However, zooming in to specific concentric shells of G3P or DHAP, where we do have enough statistics to quantify diffusion, we see that the diffusion coefficients fall rapidly as metabolites approach and transit the shell (Figure 11A), with the minimum occurring roughly where the narrowest point of the shell pores would be. The reduction in the diffusivity by more than an order of magnitude is very different from the trends previously seen in Figure 6, where the diffusivity only decreased by about a factor of 2 inside the shell. Taking into account the 10^–7^ conversion factor between Å^2^/ns and cm^2^/s, the REUS diffusion estimate in dilute solution (Figure 6) is about 10 times faster compared to the estimate from equilibrium simulations in high metabolite concentrations (Figure 11A). As we see below in closer analysis for the protein motions in this confined space, the origins for this difference may just be due to different viscosities in the two simulated solutions.

**Figure 11:**
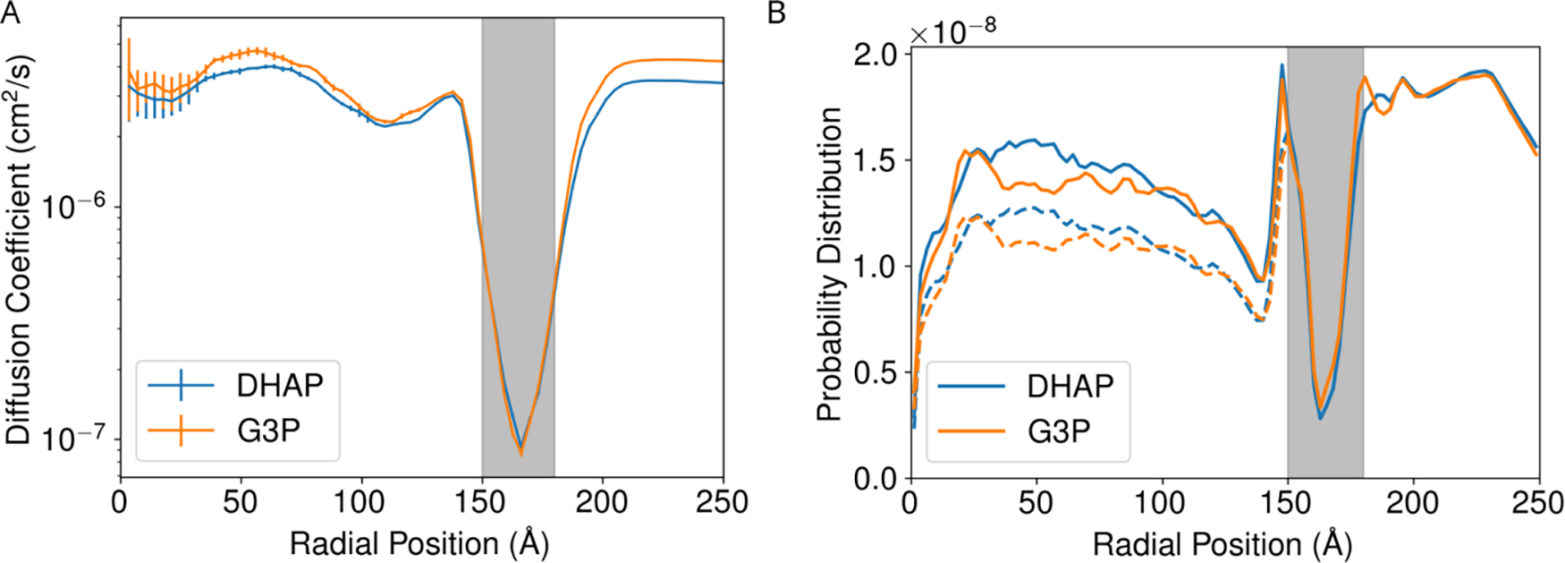
(A) An alternative view of Figure 10B for the small molecule metabolites, showing diffusion coefficients for metabolites depending on their distance from the center of the BMC, highlighting the shell protein region where diffusion is slowest. To facilitate computation, diffusion was calculated by binning small molecules according to their radial position, and then tracking their displacement over 1 ns intervals. Error estimates are determined from the standard deviation between the estimates from the three independent trajectories. Since fewer molecules are found for smaller radial values, the uncertainties in the diffusion coefficients are larger. (B) Probability distribution of metabolites per area along the radius in all molecular trajectories. The solid lines are for the probability where we consider the free diffusion volume and dashed line is against the entire interior volume regardless of protein occupancy. Inside the shell we are assuming that the 20% of the area would be occupied by the protein and the rest is free area for the metabolite to diffuse, based on the assumptions we made in designing the simulation model.

Beyond the movement of the metabolites, there is also a notable asymmetry in the metabolite distribution. Figure 11B shows this most clearly, with dissimilar metabolite concentrations inside and outside the shell. This same concentration imbalance is also observable from Figure 9, and would lead to more outbound rather than inbound metabolites. If we break down the permeation events we tracked previously, only 2 permeation events were inbound, while the other 28 exited the shell, following the concentration gradient exactly. We also tracked the movement of ion going in and out of the BMC shell and we found that the positively charged sodium has more inbound transition (Table S1) and the negatively charged chlorine has more out outbound transition­(Table S2). For negatively charged metabolites and ions together with a negatively charged interior (Figure S35), this should not be totally surprising, but does underscore what kind of surprises are possible at the nanoscale, as we took pains to add metabolites randomly initially.

Quantifying protein motions, rather than those of the small molecules, highlights the same basic physics of diffusion within a crowded environment. From Figure 12A, we see that the relationship between the protein complex size and diffusion coefficient follows a similar trend to the Stokes–Einstein-Sutherland relationship,^77,104,105^ where $D\propto \frac{1}{r}$ . However, since some proteins within our system are multimers, and can form higher order assemblies during simulation (Figure 12B), using the protein radius for an individual monomer did not yield a particularly satisfying fit. Instead, we make the simplifying assumption that a multimer complex is approximately spherical, with its radius related to the cube root of the molecular weight for the complex and the protein density, inverting the common expression $MW=\frac{4}{3}\pi r^{3}\rho$ . Putting this together, the diffusion coefficient wouldbe

8 $$D=\frac{k_{B}T}{6\pi \eta }\sqrt[{3}]{\frac{4\pi \rho }{3MW}}$$

Given our simulated temperature (298 K), assuming a protein density (ρ) of 1.35 g/cm^3^,^106^ and accounting for the appropriate unit conversions, eq 8 would predict diffusion coefficients of approximately 90 × 10^–8^cm^2^/s for a 50 kDa protein complex in neat solution where the viscosity η  ≈  1 cP. Our observed diffusion coefficients in Figure 12A are far lower than this value, suggesting that the viscosity is nearly 9 times higher within the crowded BMC shell. Values for η near 10 cP are similar to modern estimates for viscosity for small proteins within the *E. coli* cytoplasm.^107,108^ This should not be a surprising finding, as we are intentionally modeling a BMC system that has adventitiously captured protein cargo,^35^ and so to a first approximation the contents of the BMC shell should be identical to the bacterial cytoplasm.

**Figure 12:**
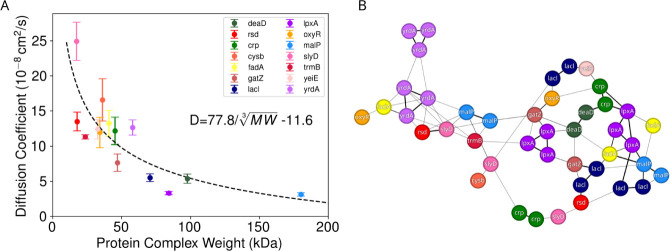
(A) Diffusion coefficient plotted against the complex weight of captured protein cargo. The curve is fit via eq 8, with an additional constant term. (B) Interaction network of captured proteins. Each peptide is color coded according to the legend in A, consistent with Figure 1. Note that the interaction network shown here represents the weighted average over all structures observed in simulation, with thicker lines for shorter distance connections.

An interesting wrinkle from Figure 12A is that we add an additional constant term to eq 8 when we do the fit. While we do not show alternative fits here, removing the constant term and fitting the relationship shown in Figure 12A to a power law with the form *D* = *AMW* ^α^ yields $\alpha ∼\frac{2}{3}$ . In our view, there is no reasonable physical justification to assume $\alpha =\frac{2}{3}$ . If we force $\alpha =\frac{1}{3}$ without allowing for a constant offset, the fit is very poor, unable to accurately capture both high diffusivity at low molecular weights and lower diffusion coefficients at higher molecular weight. We think the offset in the fit reported in Figure 12A is to correct the underlying assumption we made in eq 8, namely that the density of a protein is constant. Protein density varies with molecular weight,^106^ with lighter proteins being denser than our assumption for a constant protein density. This would mean that the diffusion for these lighter proteins is faster than the simple model from eq 8, and we need a negative constant offset, as well as a larger fitting coefficient to correct for this.

### Protein Interaction Network Formation and Evolution

Another way to visualize the crowding within the BMC shell is to explicitly map contacts between individual proteins over time. In the Supporting Animations S1–S3, we can track interactions between the loaded protein cargos explicitly over time. An interaction network between the proteins forms spontaneously within the confined space, with the final connectivity shown in Figure 12B. Some of these interactions are due to monomer stoichiometry, as dimers and trimers remain intact during the short simulations here. Spontaneous network formation has been observed before as well in prior large scale simulations.^43^ As with that prior work, there is no reason to suspect that the network formation is essential to protein function, since the proteins here are encapsulated adventitiously during BMC formation. We suspect that this spontaneous protein network formation is driven entropically, as the water molecules released from the nearest solvation shell when proteins directly interact can join the bulk and can explore rotational and translational degrees of freedom.

A different way to track network formation is to consider how the nearest neighbors for individual proteins change over time as the cargo proteins cluster through liquid–liquid phase separation. Initially, the distribution of nearest neighbors tilts towards longer distances at the beginning of the simulation, while at the end the formation of these contacts reduces the distance of closest approach (Figure 13). Comparing with similar models, where the proteins are membrane embedded,^43^ the typical distance between nearest neighbors shown in Figure 13 is shorter. This is to be expected when interactions can be made in all three directions, rather than confined to the two-dimensional membrane surface. However, it is still striking that the 10th nearest protein is some 20–40% closer in 3D than in 2D. The compactness of cargo protein can also be seen from the snapshot extracted from the simulation trajectory (Figure S37). Looking at the supplementary animations, we can say that the network connectivity is increasing as the simulation goes on and the cargo comes closer together which is depicted in the snapshot animating along the interaction network. From Figure 13, we do see that longer simulations may be required to fully form the networks, as the nearest neighbor distances continue to decrease as a function of time.

**Figure 13:**
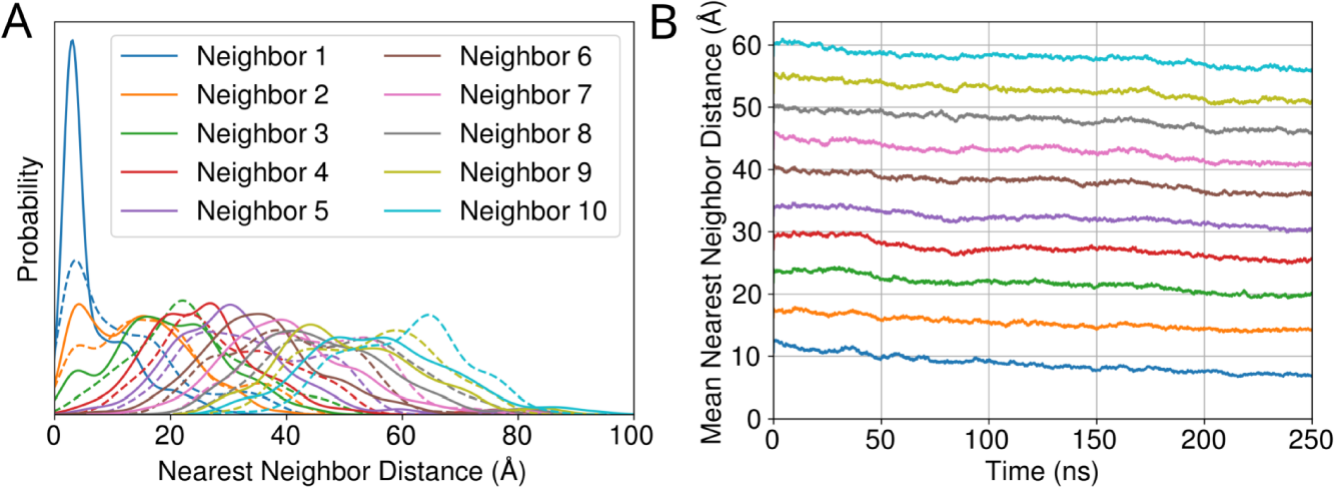
Nearest neighbor analysis for the cargo proteins within the BMC shell. In (A), we report the distribution for the 10 nearest neighbors both at the beginning (dashed lines), and at the end (solid lines) of the simulation. In (B), we show the mean distance for individual nearest neighbors across all cargo proteins and across all three simulation replicates over the 250 ns simulation time.

The contact evolution and diffusion analysis from Figures 12 and 13 totally ignore the shell proteins themselves, and are focused only on the protein cargo within the BMC shell. In part, this is done for expediency. The shell proteins are not independent from one another, and move as a unit within our simulations through rotational and translational degrees of freedom. However, this motion is incredibly slow on the time scales we are simulating, as the full BMC shell is much larger than a single protein. To a first approximation, the full shell would be expected to diffuse following a Stokes–Einstein formalism, and expressions similar to eq 8 would be suitable to express its diffusion coefficient. The challenge for us is that because the shell is so large, with a radius of approximately 180 Å, that the mean displacement for the whole shell is anticipated to be only 40 Å or so over 250 ns in neat water. In practice, the high concentration of DHAP and G3P in solution means that the environment the HO shell is diffusing in is more viscous than we would expect, and so the displacements we actually observe are closer to 3 Å over this time scale (Figure S38). The wide variation in for the displacement over time increases the uncertainty for the diffusion coefficient sufficiently that we choose not to report one here.

The final comparison we would like to make from this really unique simulation system are the protein–protein contacts between the shell and its cargo, and internal contacts among the cargo (Figure 14). We do not know what the relative number of contacts should be *a priori* to direct biomolecular condensate formation within the BMC, but for this particular BMC shell geometry with adventitiously loaded cargo, there are roughly equally many contacts between the shell and the cargo and between the cargo itself. Naturally, this kind of relationship will be related to the size of the BMC shell, with larger shells tending to feature more cargo/cargo interactions while cargoes loaded into smaller shells will have more interactions with the shell surface. Thus, larger BMCs like carboxysomes can feature directional packing of their rubisco cargo based on cargo/cargo interactions, as has been observed by cryo electron tomography^109–111^ while packing smaller shells like the HO shell studied here may be governed more by surface effects while encapsulating novel catalytic pathways.

**Figure 14:**
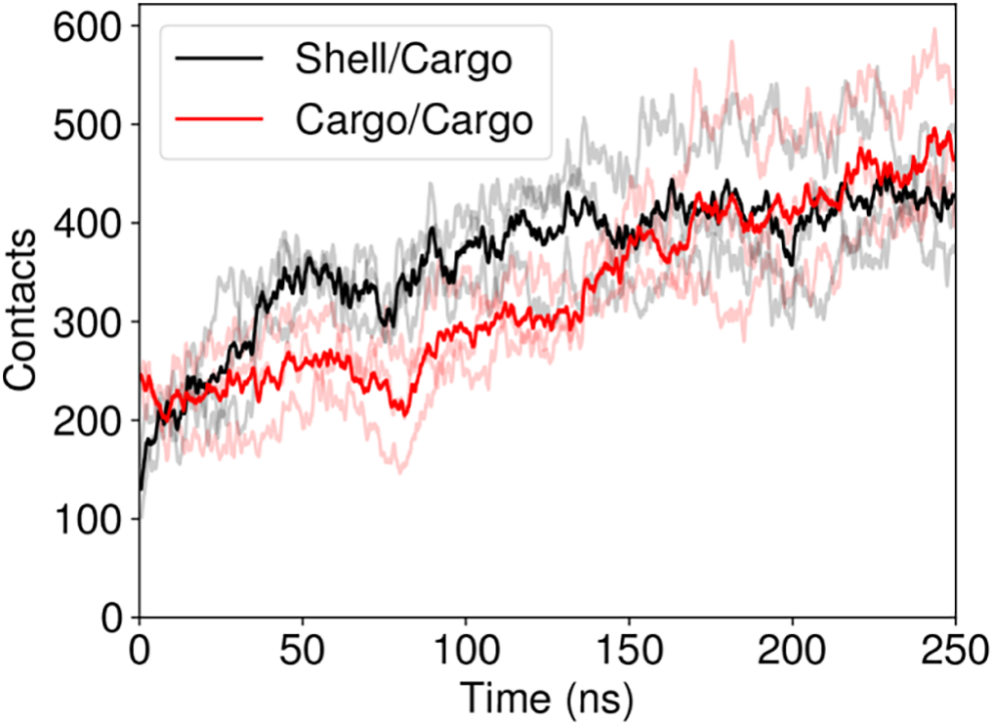
Contact comparison between the total number of shell/cargo protein contacts and cargo/cargo protein contacts. The contacts are quantified through eq 1, and contacts between multimers are excluded from the overall sum. The darker red and black lines indicate the average from across the three simulations, while the three individual simulation traces are shown in a lighter shade.

## Conclusion

BMCs share alot of commonalities with some viral envelopes, and as a result share many challenges to simulation. An atomic-scale system for an intact BMC can be very big, however advances in computing hardware continues to drive down the cost of simulating these large systems. Indeed, by estimating the total electricity cost associated with the simulations described in this paper, the energy intensity for running these calculations continues to decline with each advancing hardware generation, and so we anticipate that the kinds of large scale simulations we performed here will become more common in the coming years.

We leverage these advances in computing hardware to simulate both an intact BMC shell and a planar BMC shell fragment, deriving two independent estimates for permeability through the shell. While permeabilities determined via the two independent methods differed by more than 2 order of magnitude, the overall picture for both estimates is the same. The high estimated permeability suggests that the concentrations inside and outside the BMC shells will be similar under most circumstances, and that the *tpi* enzyme specifically will have no issues obtaining its substrates once encapsulated. We suspect, based on similar permeability coefficients for other small molecules through BMCs,^5^ that this may be a larger trend in BMCs, where so long as a metabolite can physically fit through the pores within the BMC, the permeability coefficients will be reasonably high until the metabolite concentration is so high that metabolites are limited by transiting the pores one by one.

Analyzing the larger simulation for additional physical insight was also fruitful, delivering an estimate for the viscosity in a packed BMC, which is an improvement on prior simulations that only had solution inside. The cargo proteins reduce diffusion beyond what the high metabolite concentration does alone. While our simulations are too short to be fully converged, we do see some evidence for spontaneous protein aggregation and clustering within the BMC shell. Seeing liquid–liquid phase separation for encapsulated proteins spontaneously in simulation gives us greater confidence that this kind of condensation is not only key to normal BMC function, but will continue to be a fruitful research direction as we engineer new applications for BMCs.^112–114^ Such molecular level insight would be really difficult to obtain without simulation, and highlights the impact simulation can have on designing nanostructures for (bio)­engineering purposes.

## Supplementary Material









## Data Availability

All input scripts to build and run molecular simulations are made publicly available on Zenodo https://doi.org/10.5281/zenodo.15776380.

## Notes

Cargo proteins intra interaction during the MD simulation­(left) and snapshot of MD simualtion for the same time step (right) for replica 1 (MP4)
Cargo proteins intra interaction during the MD simulation­(left) and snapshot of MD simualtion for the same time step (right) for replica 2 (MP4)
Cargo proteins intra interaction during the MD simulation­(left) and snapshot of MD simualtion for the same time step (right) for replica 3 (MP4)
Tables S1 and S2: Transition events for sodium and chlorine ion in the simulation trajectory; Figures S1–S37: Traces for trace for DHAP molecules (PDF)

## #

School of Chemistry, University College Dublin, Dublin, Ireland
