# Supplementary material for: Molecular Modeling and Molecular Dynamics Simulation of a Packed and Intact Bacterial Microcompartment
Source: J Phys Chem B. 2025 Nov 5;129(50):12811–27. doi: 10.1021/acs.jpcb.5c05178 (PMC12720247; doi:10.1021/acs.jpcb.5c05178)
Supplement: Supplementary file 4 [file jp5c05178_si_004.pdf]

Supporting Information: Molecular Modeling and Molecular Dynamics Simulation of a Packed and Intact Bacterial Microcompartment

Saad Raza,[†] Neetu Singh Yadav,[†] Alexander Jussupow,[‡,¶] Cheryl A. Kerfeld,[§,†,‖,⊥] Michael Feig,[‡] and Josh V. Vermaas[*,†,‡]

†*MSU-DOE Plant Research Laboratory, College of Natural Science, Michigan State University, East Lansing MI 48824*

‡*Department Of Biochemistry and Molecular Biology, College of Natural Science, Michigan State University, East Lansing MI 48824*

¶*Current affiliation: School of Chemistry, University College Dublin, Dublin, Ireland*

§*Environmental Genomics and Systems Biology Division, Lawrence Berkeley National Laboratory, Berkeley, California 94702*

‖*Biochemistry and Molecular Biology Department, Michigan State University, East Lansing, Michigan 48824*

⊥*Molecular Biophysics and Integrated Bioimaging Division, Lawrence Berkeley National Laboratory, Berkeley, California 94702*

E-mail: vermaasj@msu.edu

Phone: +1 (517) 884-6937

Supplementary Animation 1 Cargo proteins intra interaction during the MD simulation (left) and snapshot of MD simualtion for the same time step (right) for replica 1. The cargo proteins are color coded according to the names in the interaction network plot. The protein are represented as surfaces, shell proteins are shown as transparent and cargo proteins as opaque chalky surface. Trimers are color coded in shades of purple, dimers are in the shades of blue and green and monomers are color coded in shades of red, yellow and orange.

Supplementary Animation 2 Cargo proteins intra interaction during the MD simulation (left) and snapshot of MD simualtion for the same time step (right) for replica 2. The cargo proteins are color coded according to the names in the interaction network plot. The protein are represented as surfaces, shell proteins are shown as transparent and cargo proteins as opaque chalky surface. Trimers are color coded in shades of purple, dimers are in the shades of blue and green and monomers are color coded in shades of red, yellow and orange.

Supplementary Animation 3 Cargo proteins intra interaction during the MD simulation (left) and snapshot of MD simualtion for the same time step (right) for replica 3. The cargo proteins are color coded according to the names in the interaction network plot. The protein are represented as surfaces, shell proteins are shown as transparent and cargo proteins as opaque chalky surface. Trimers are color coded in shades of purple, dimers are in the shades of blue and green and monomers are color coded in shades of red, yellow and orange.

Table S1: Transition event for sodium ion in the simulation trajectory.

Replica	Total Transitions	Inside to Outside	Outside to Inside
Run1	564	139	425
Run2	667	180	487
Run3	495	106	389

Table S2: Transition event for chlorine ion in the simulation trajectory.

Replica	Total Transitions	Inside to Outside	Outside to Inside
Run1	115	86	29
Run2	111	79	32
Run3	125	87	38

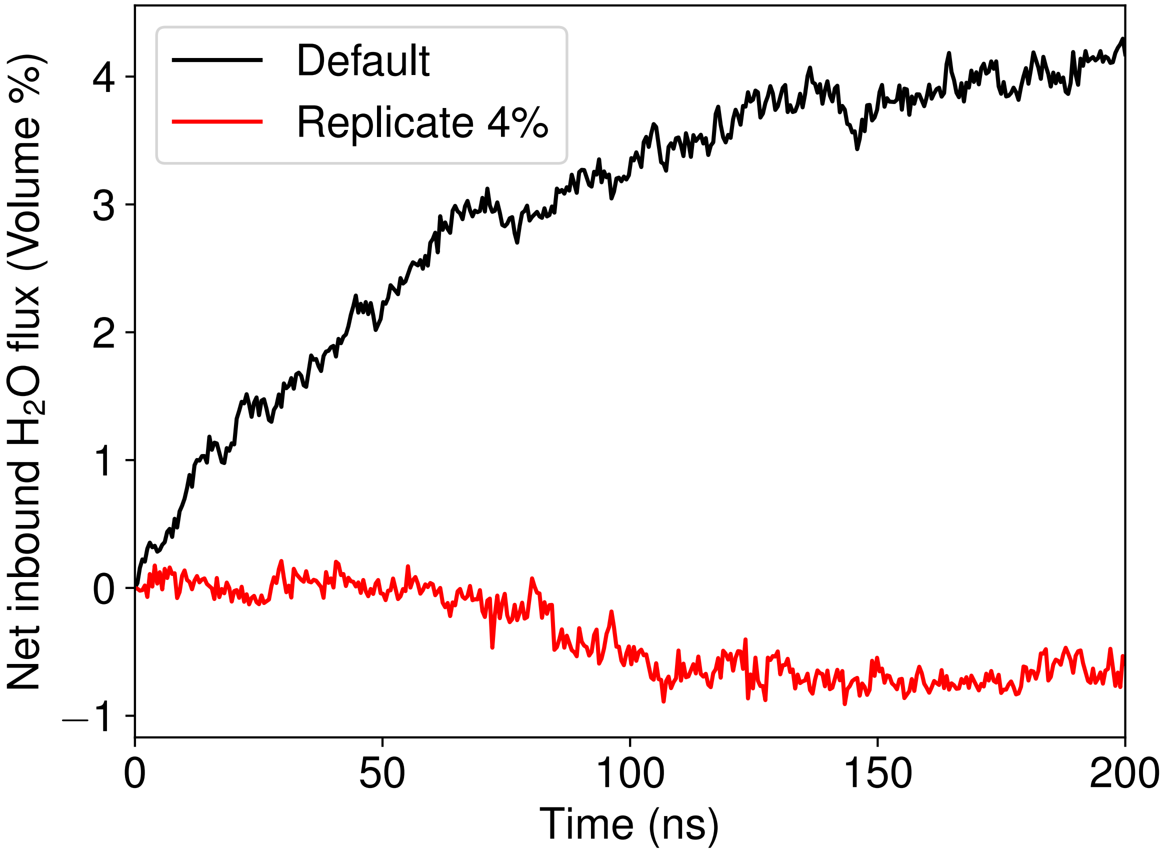

Figure S1: Net inbound water flux across an empty HO shell (simulation reported in Zuo et al 2025,[S1] both with the default VMD solvation script (black), and once after some water internal water molecules were replicated and shifted to better balance osmotic pressure.

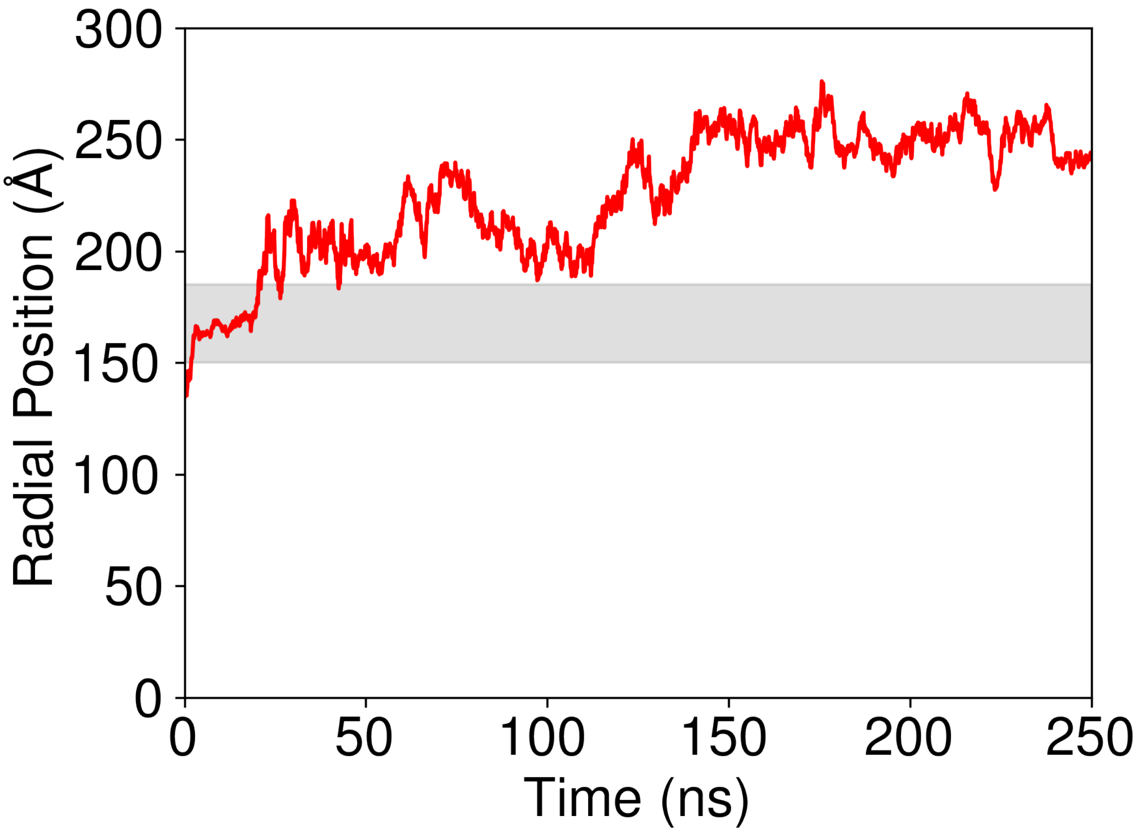

Figure S2: This is a trace for a DHAP molecule crossing the BMC shell in simulation replica 1. The gray region indicates where the shell protein is.

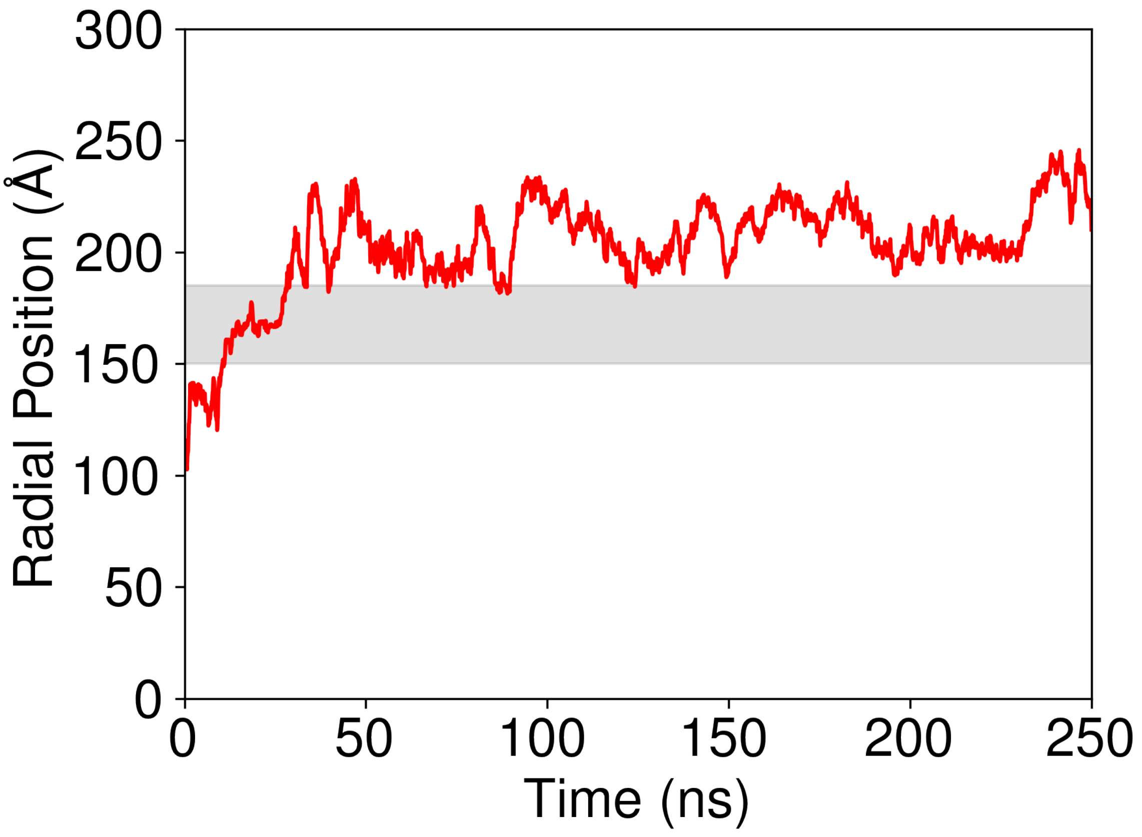

Figure S3: This is a trace for a DHAP molecule crossing the BMC shell in simulation replica 1. The gray region indicates where the shell protein is.

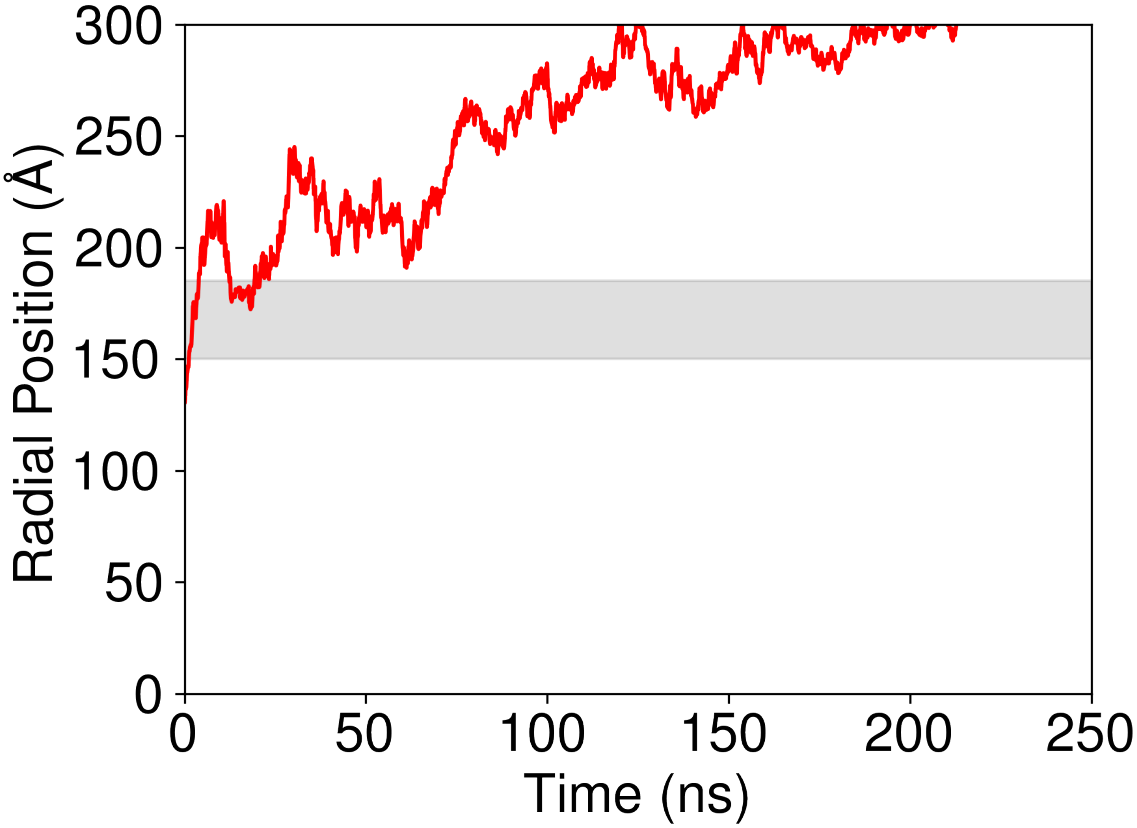

Figure S4: This is a trace for a DHAP molecule crossing the BMC shell in simulation replica 1. The gray region indicates where the shell protein is.

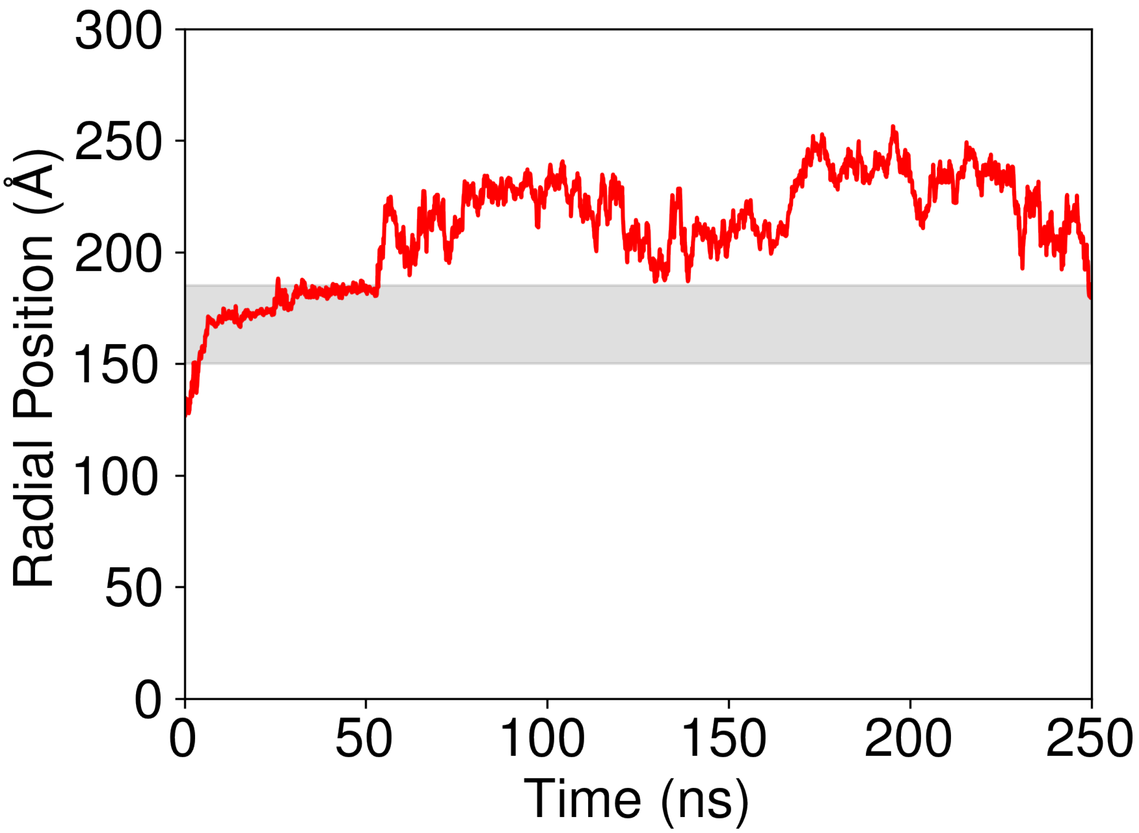

Figure S5: This is a trace for a DHAP molecule crossing the BMC shell in simulation replica 1. The gray region indicates where the shell protein is.

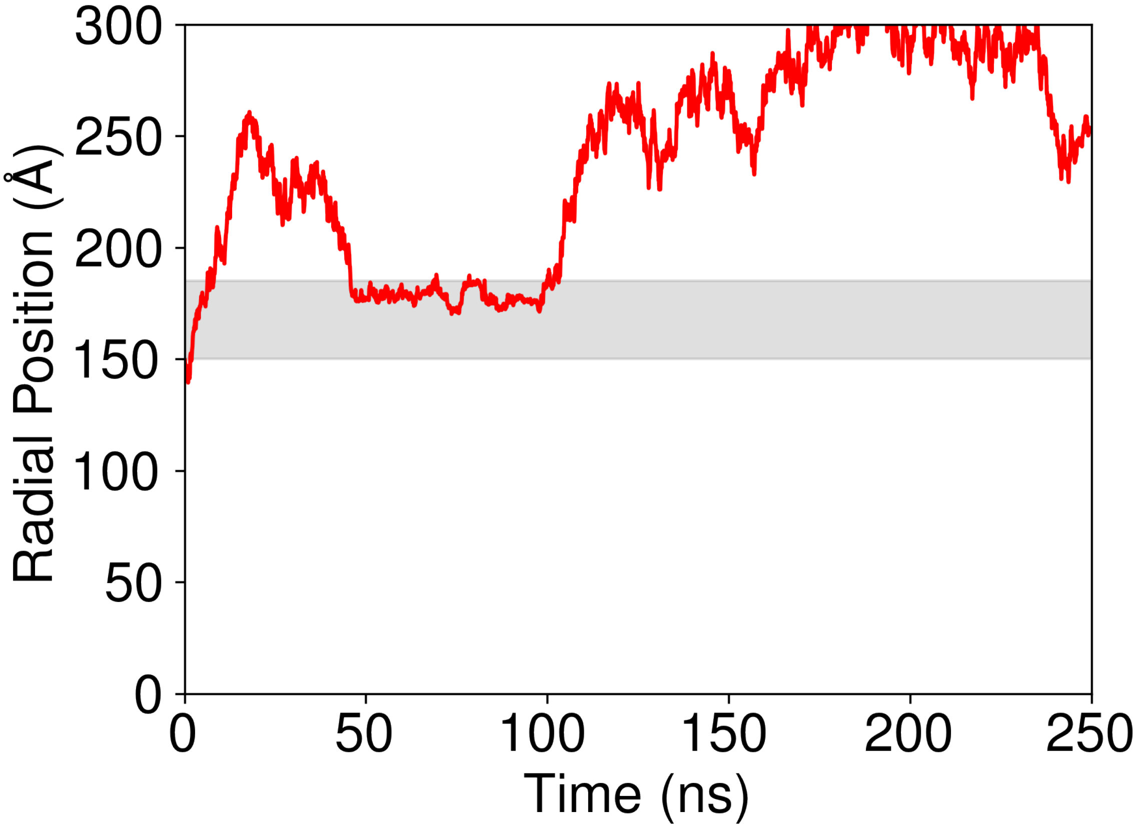

Figure S6: This is a trace for a DHAP molecule crossing the BMC shell in simulation replica 1. The gray region indicates where the shell protein is.

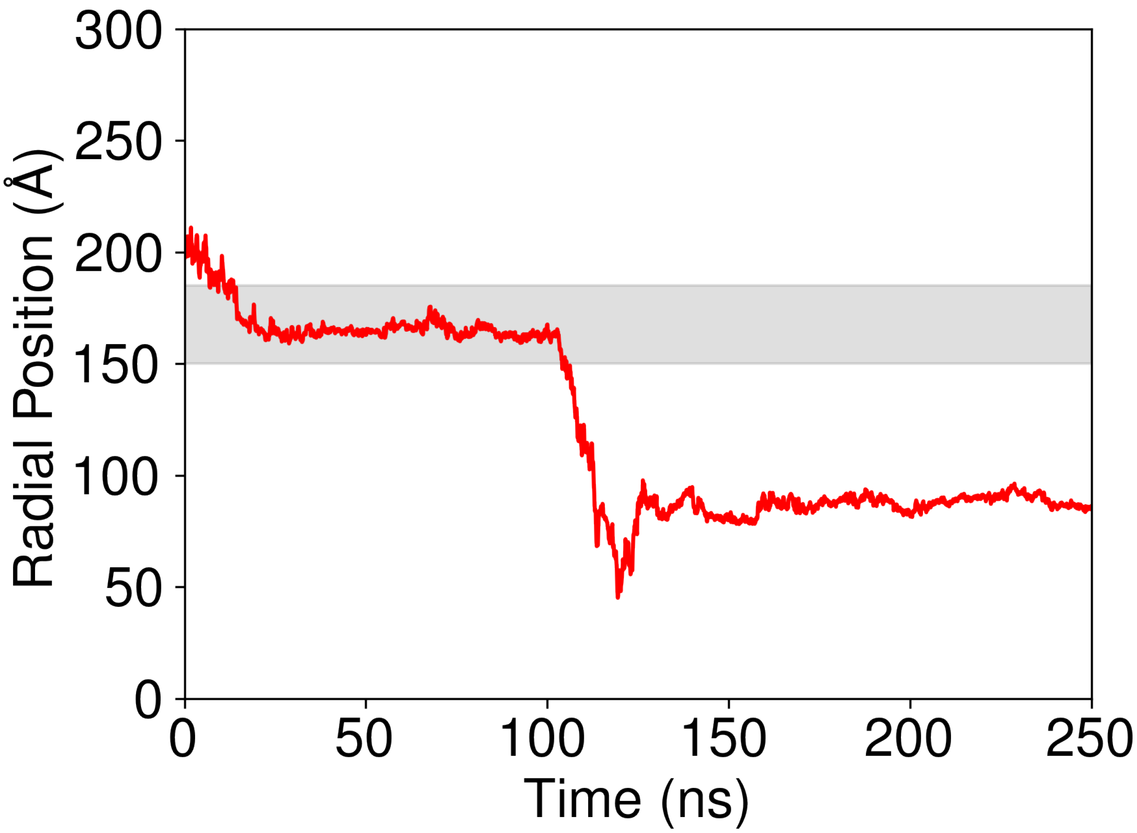

Figure S7: This is a trace for a DHAP molecule crossing the BMC shell in simulation replica 2. The gray region indicates where the shell protein is.

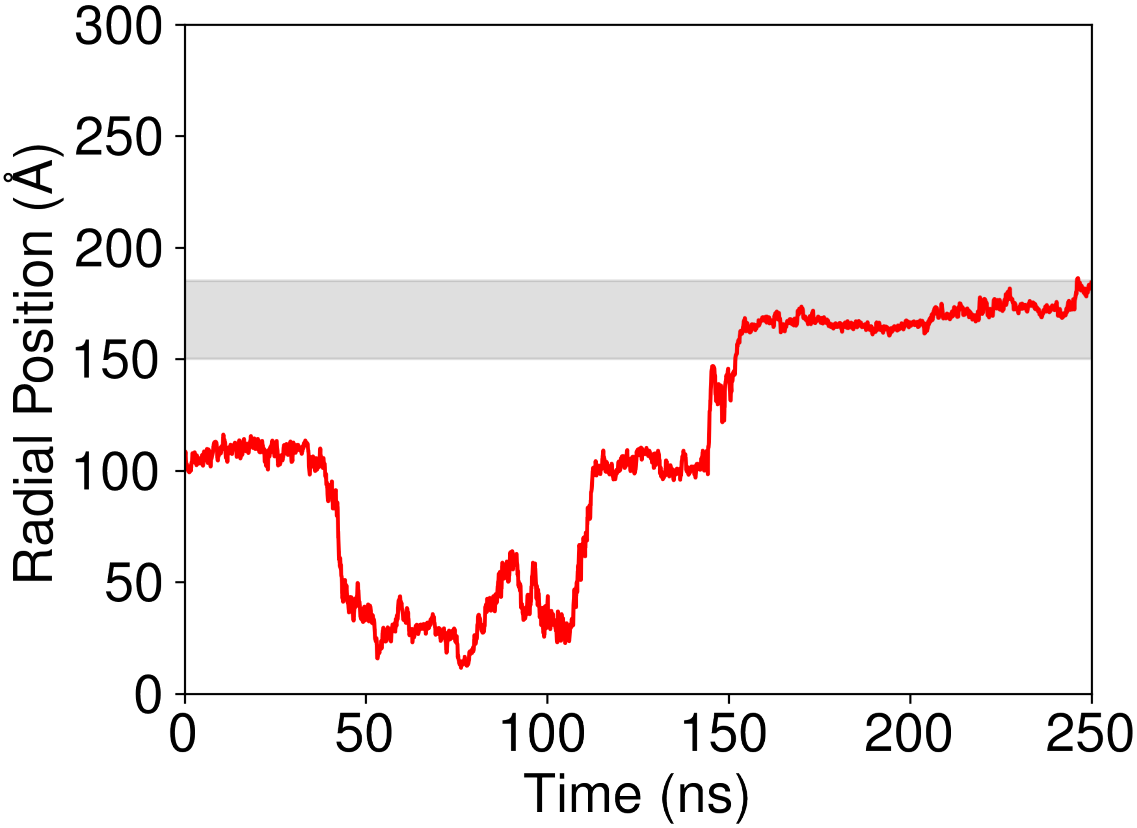

Figure S8: This is a trace for a DHAP molecule crossing the BMC shell in simulation replica 2. The gray region indicates where the shell protein is.

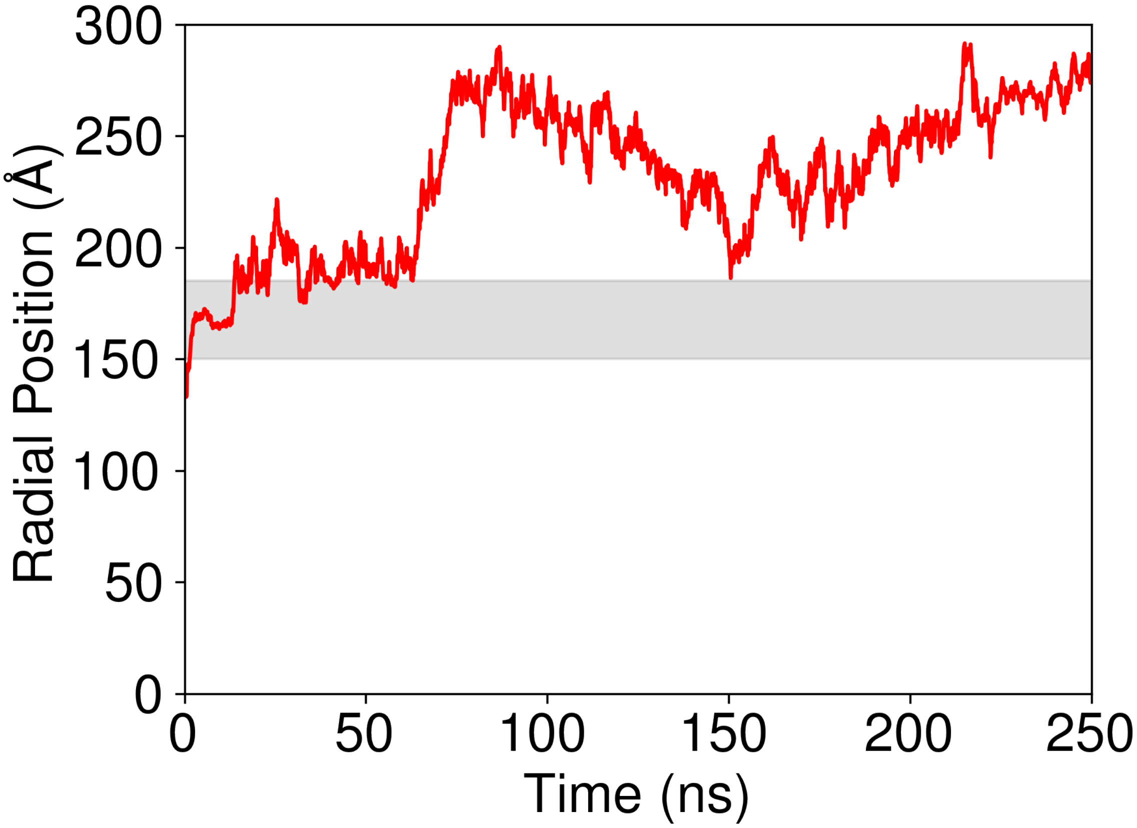

Figure S9: This is a trace for a DHAP molecule crossing the BMC shell in simulation replica 2. The gray region indicates where the shell protein is.

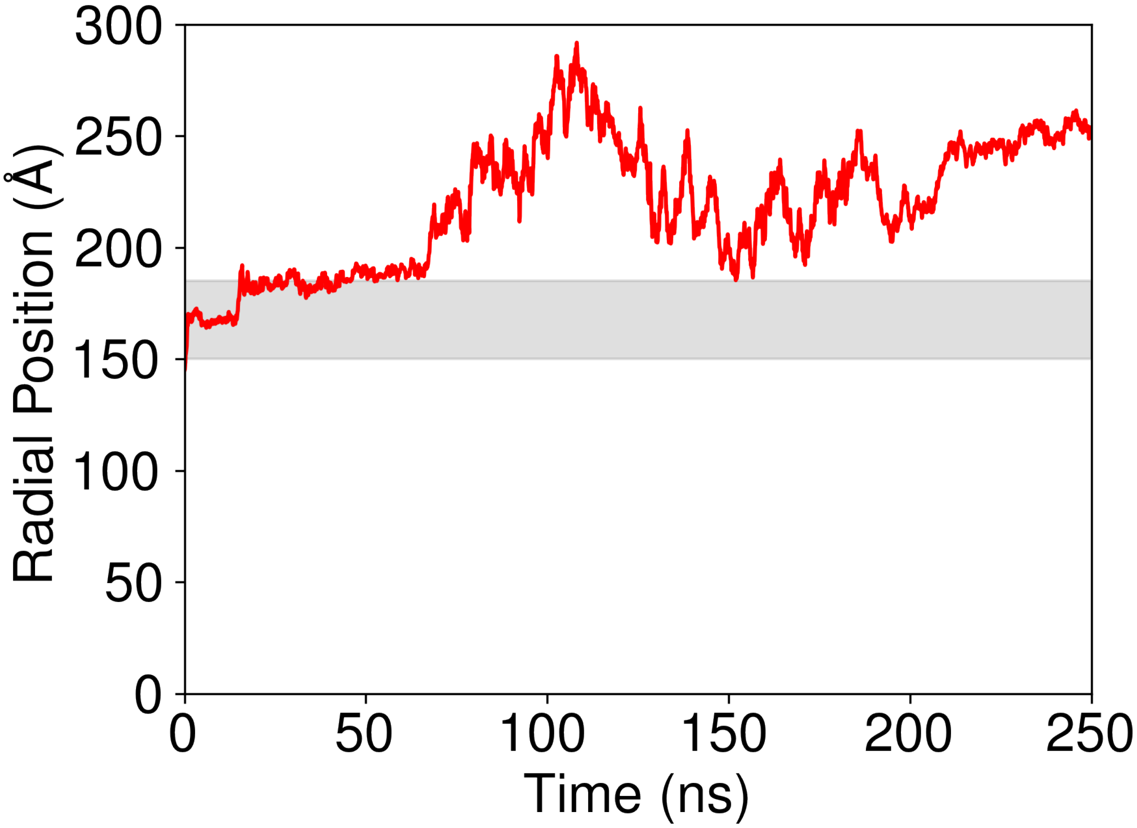

Figure S10: This is a trace for a DHAP molecule crossing the BMC shell in simulation replica 2. The gray region indicates where the shell protein is.

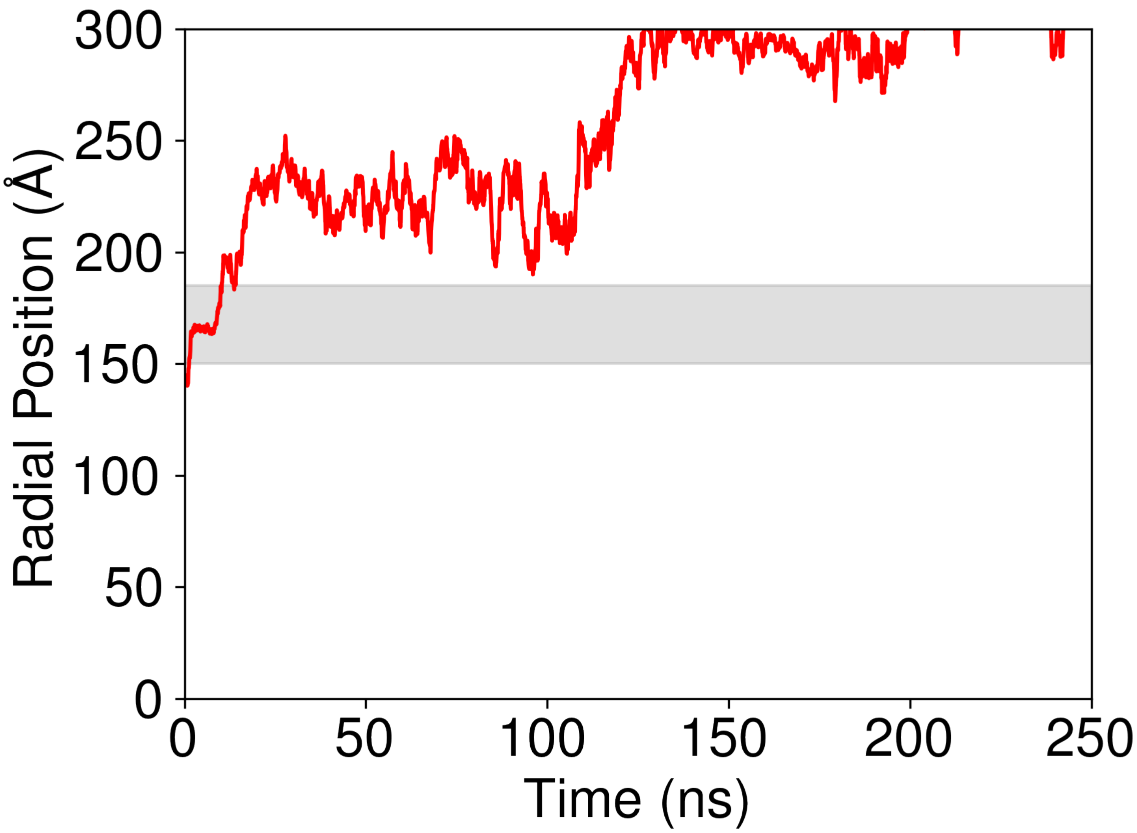

Figure S11: This is a trace for a DHAP molecule crossing the BMC shell in simulation replica 2. The gray region indicates where the shell protein is.

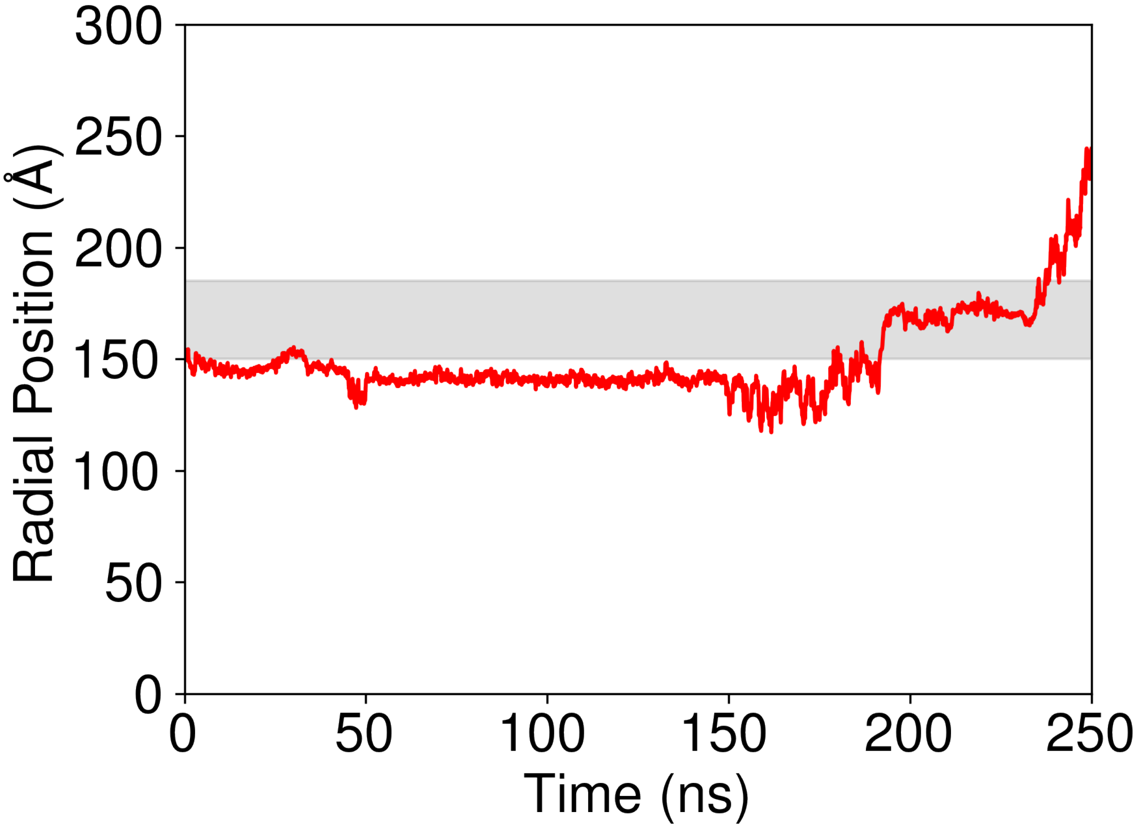

Figure S12: This is a trace for a DHAP molecule crossing the BMC shell in simulation replica 3. The gray region indicates where the shell protein is.

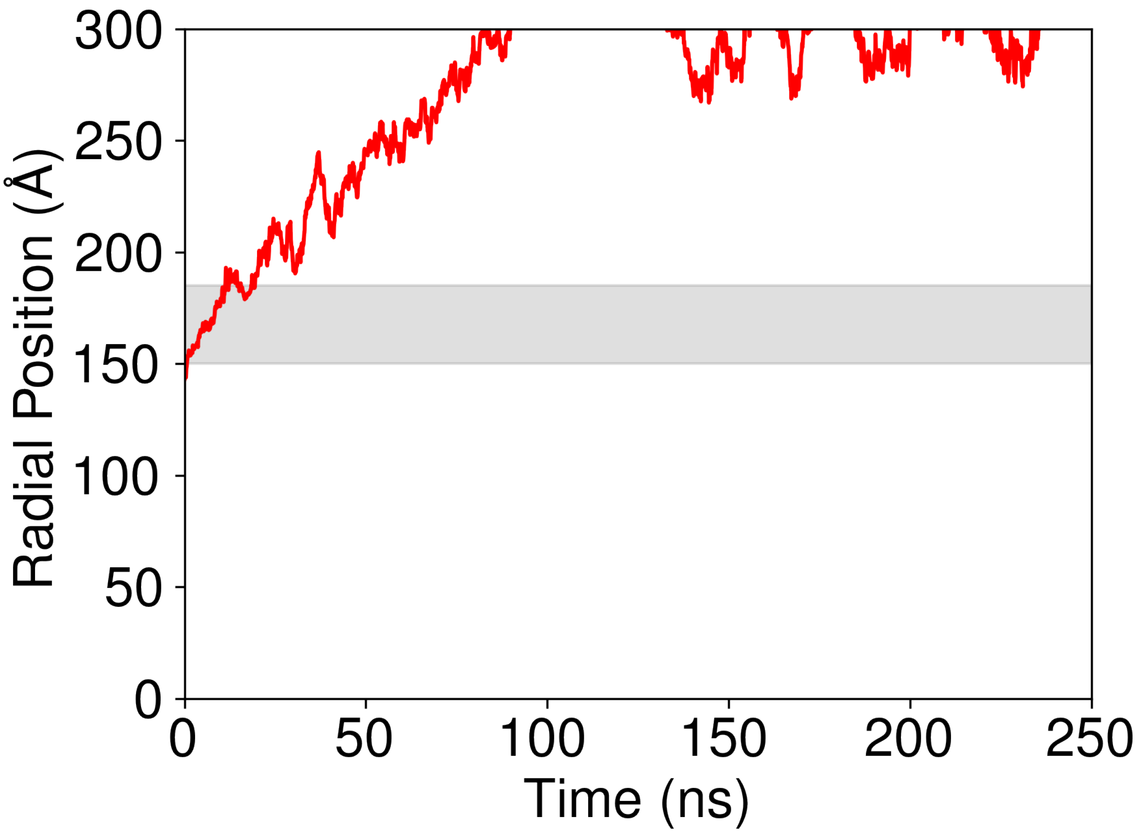

Figure S13: This is a trace for a DHAP molecule crossing the BMC shell in simulation replica 3. The gray region indicates where the shell protein is.

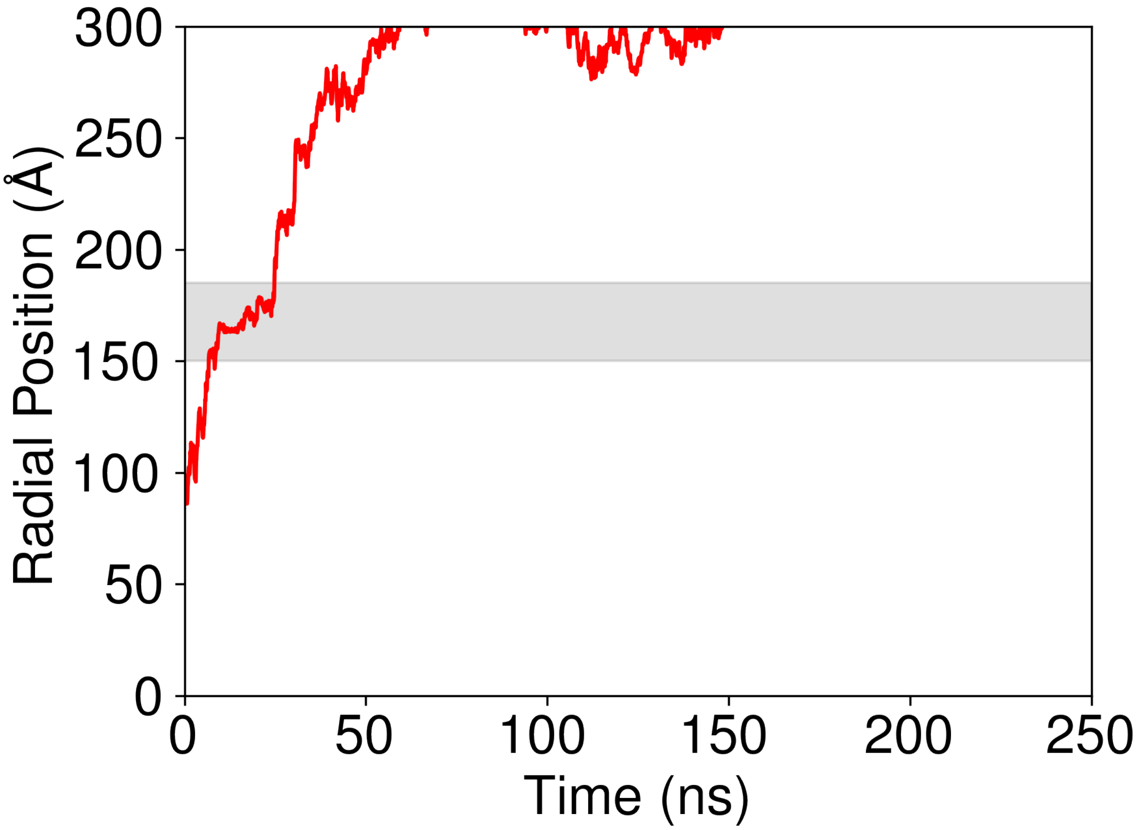

Figure S14: This is a trace for a DHAP molecule crossing the BMC shell in simulation replica 3. The gray region indicates where the shell protein is.

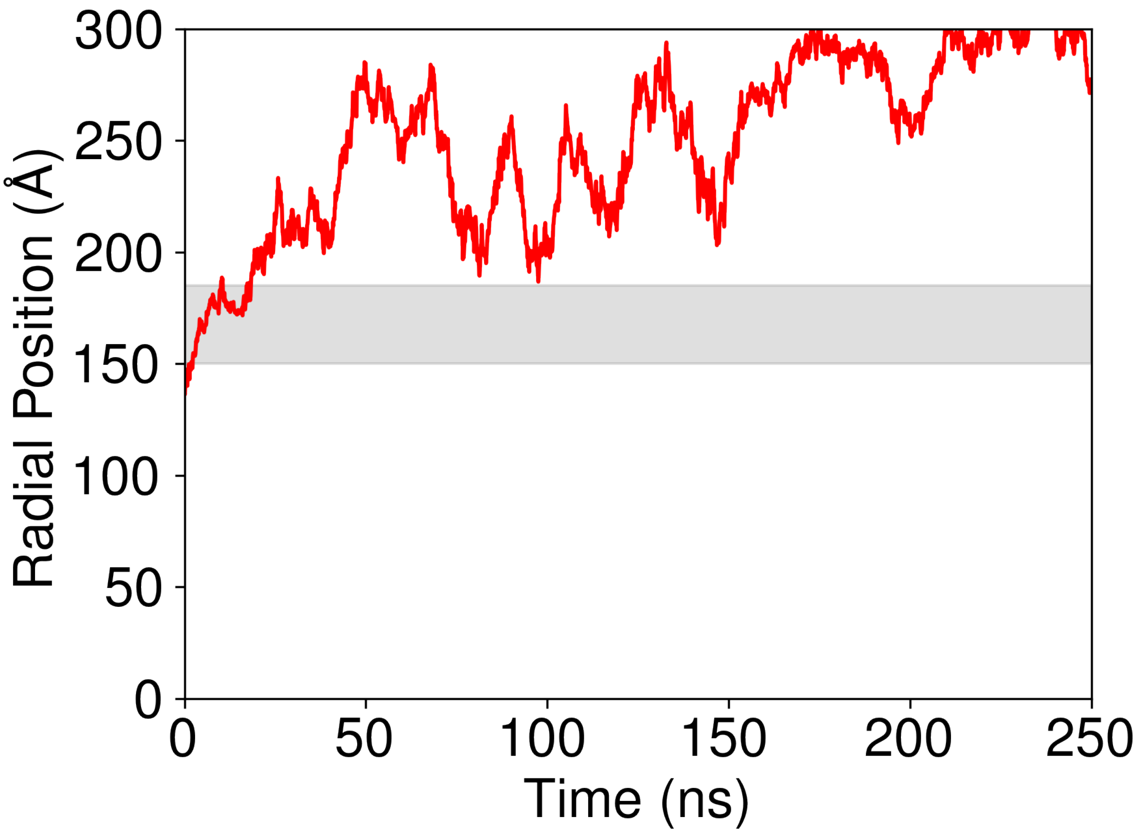

Figure S15: This is a trace for a DHAP molecule crossing the BMC shell in simulation replica 3. The gray region indicates where the shell protein is.

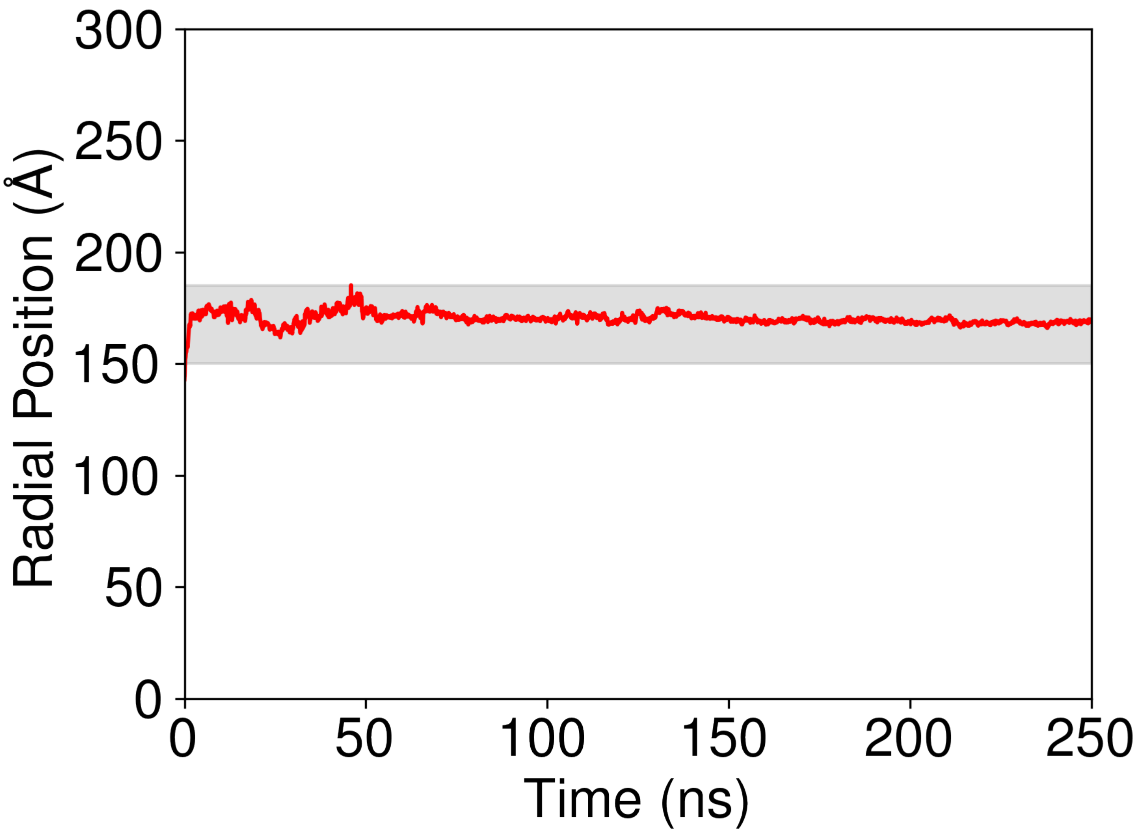

Figure S16: This is a trace for a DHAP molecule crossing the BMC shell in simulation replica 3. The gray region indicates where the shell protein is.

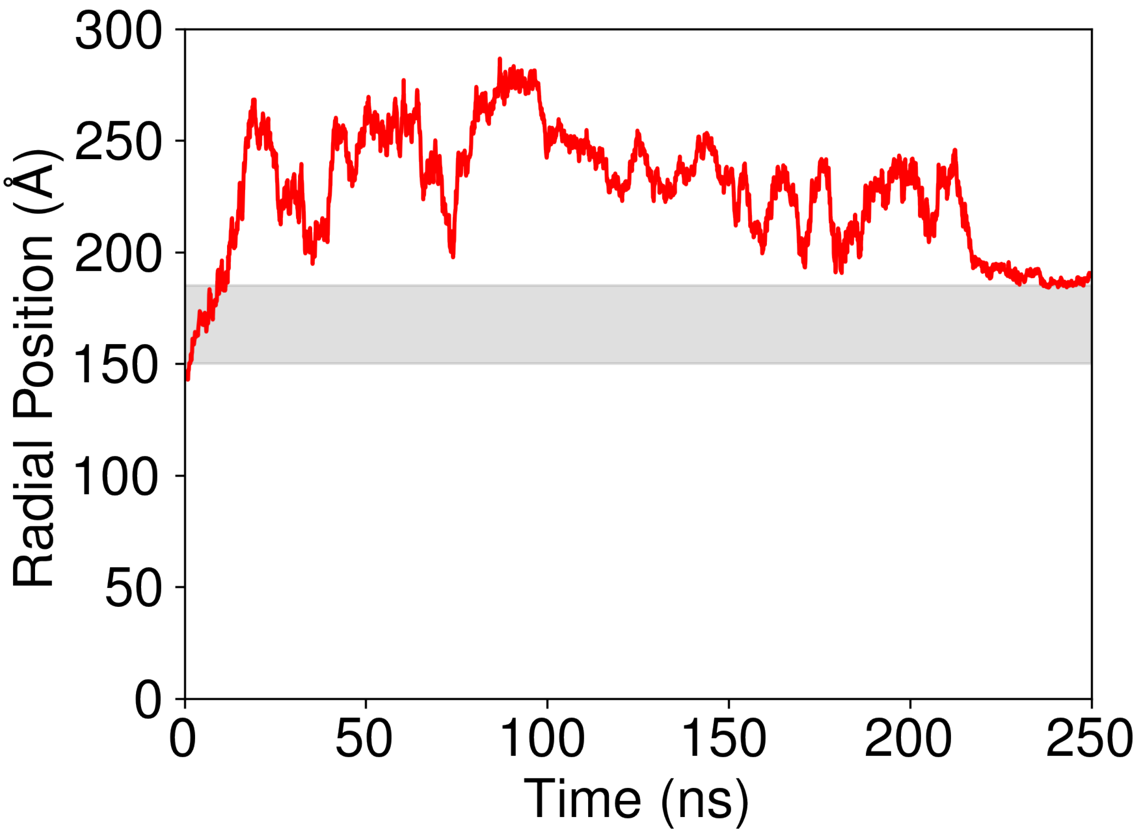

Figure S17: This is a trace for a G3P molecule crossing the BMC shell in simulation replica 1. The gray region indicates where the shell protein is.

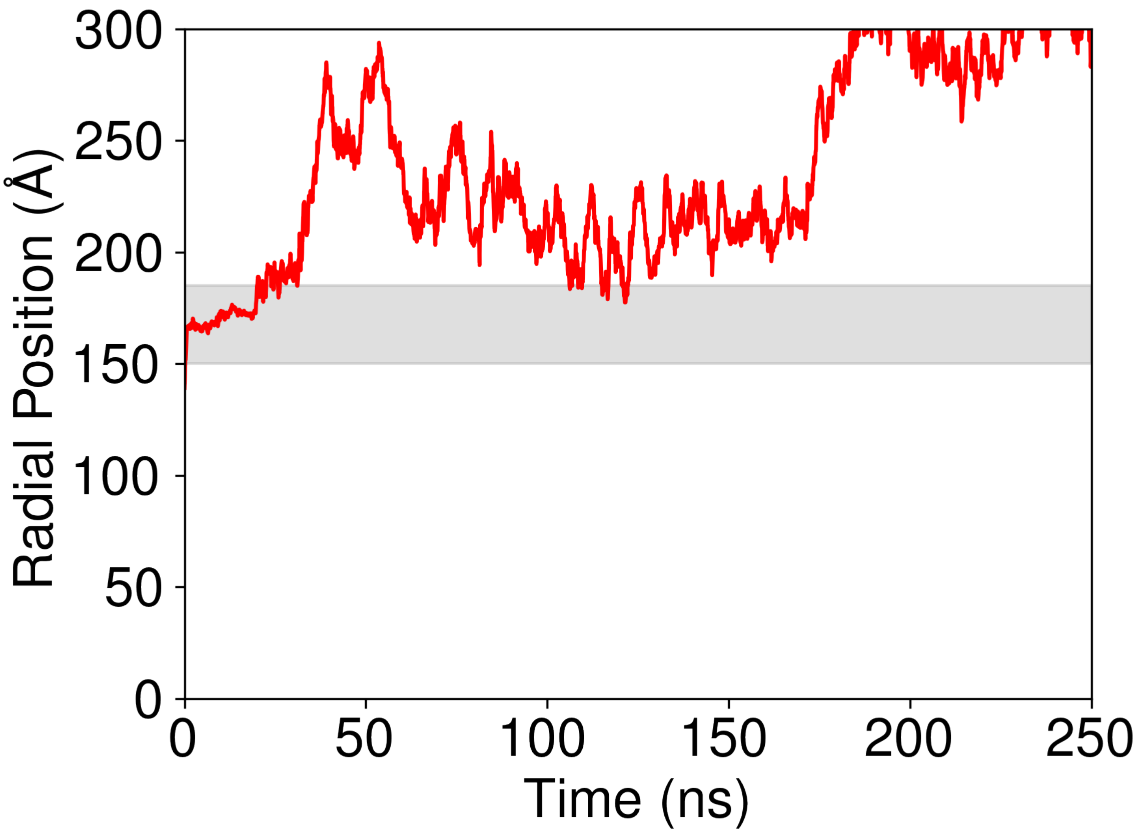

Figure S18: This is a trace for a G3P molecule crossing the BMC shell in simulation replica 1. The gray region indicates where the shell protein is.

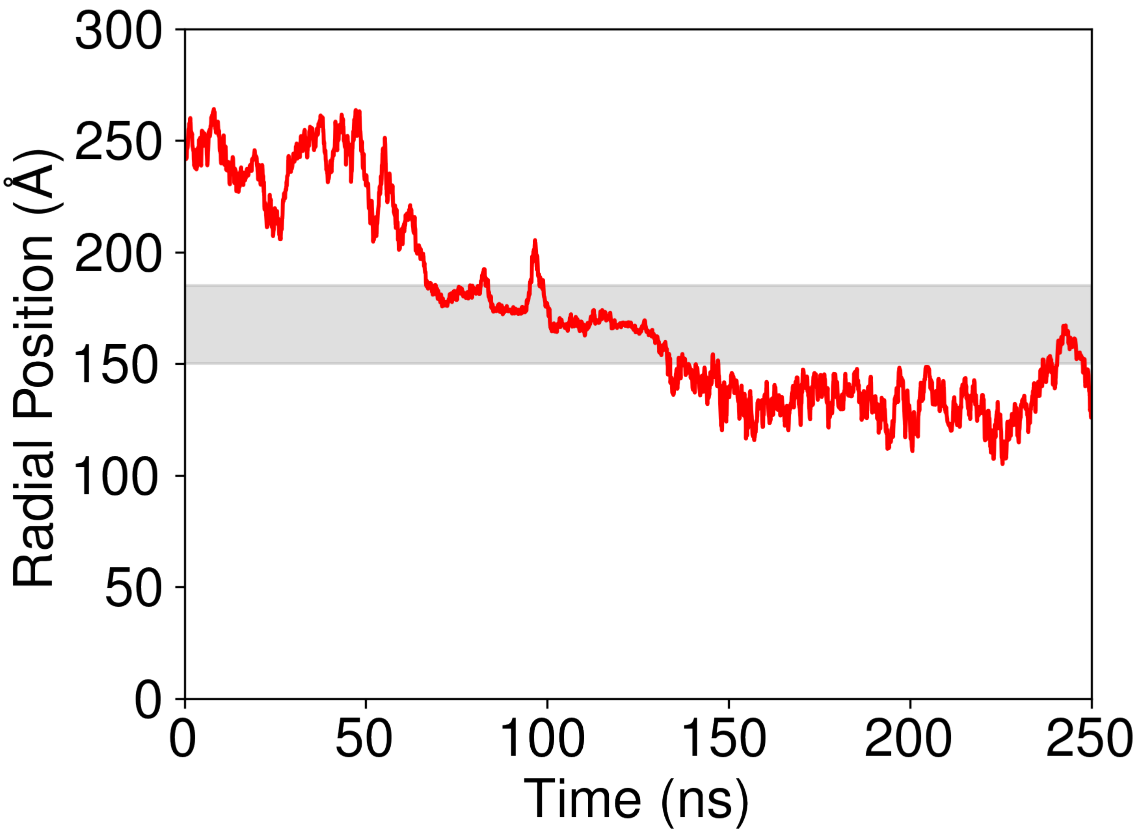

Figure S19: This is a trace for a G3P molecule crossing the BMC shell in simulation replica 2. The gray region indicates where the shell protein is.

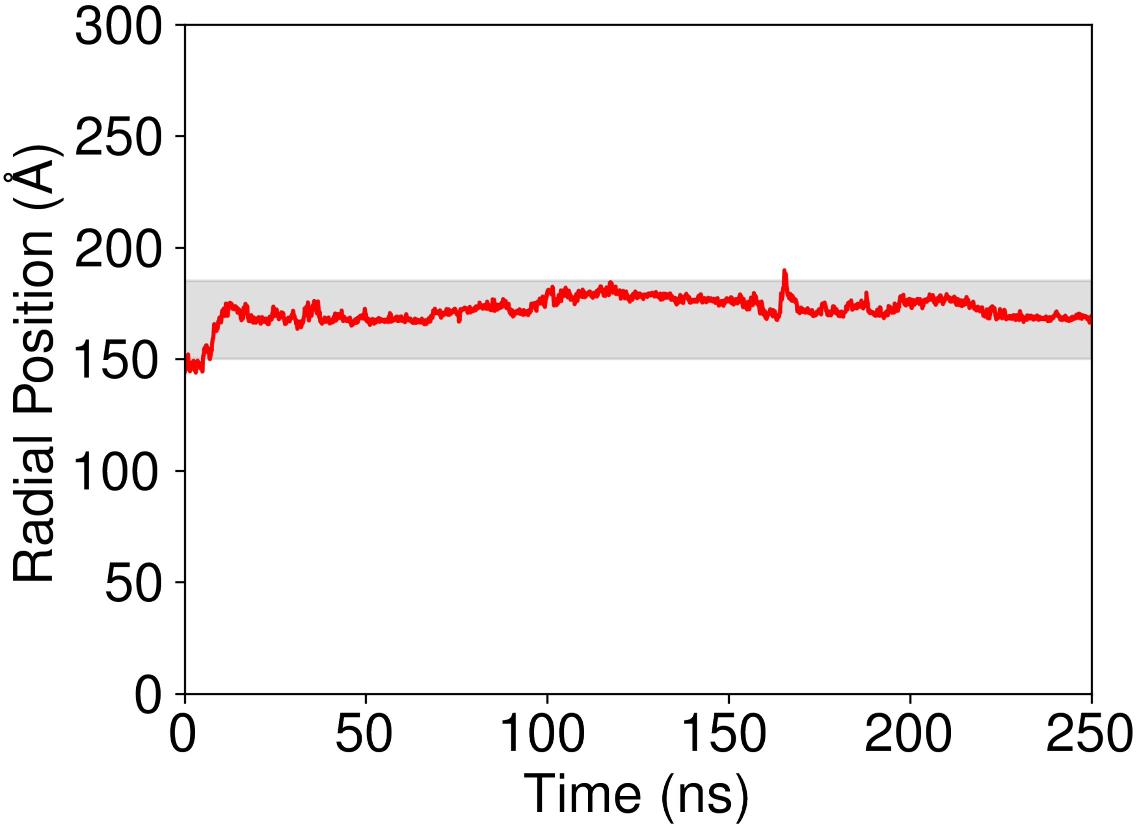

Figure S20: This is a trace for a G3P molecule crossing the BMC shell in simulation replica 2. The gray region indicates where the shell protein is.

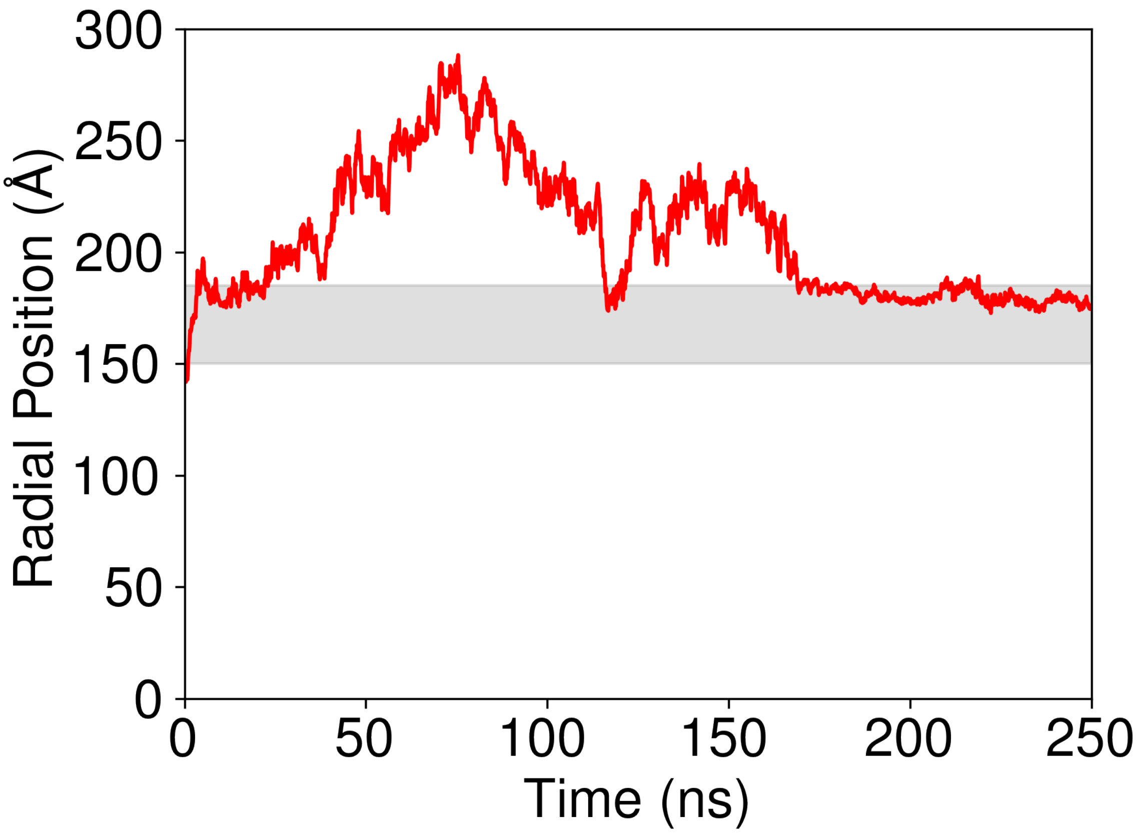

Figure S21: This is a trace for a G3P molecule crossing the BMC shell in simulation replica 2. The gray region indicates where the shell protein is.

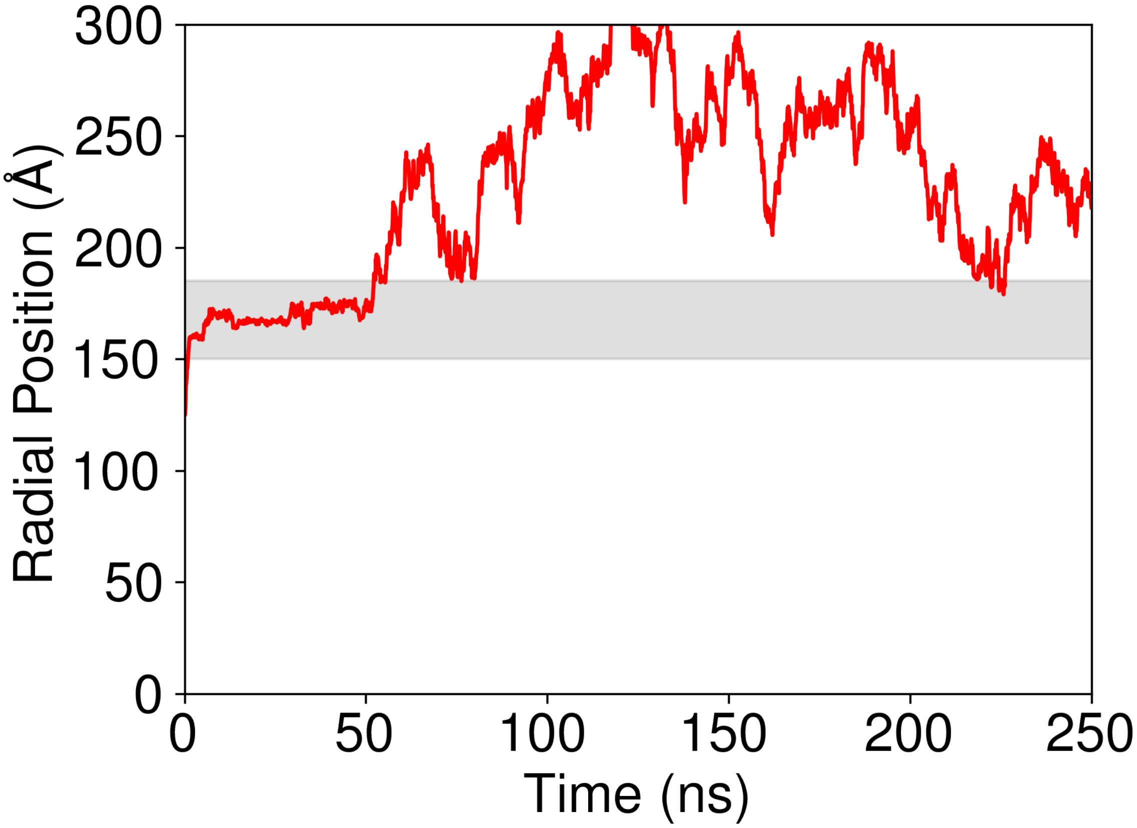

Figure S22: This is a trace for a G3P molecule crossing the BMC shell in simulation replica 2. The gray region indicates where the shell protein is.

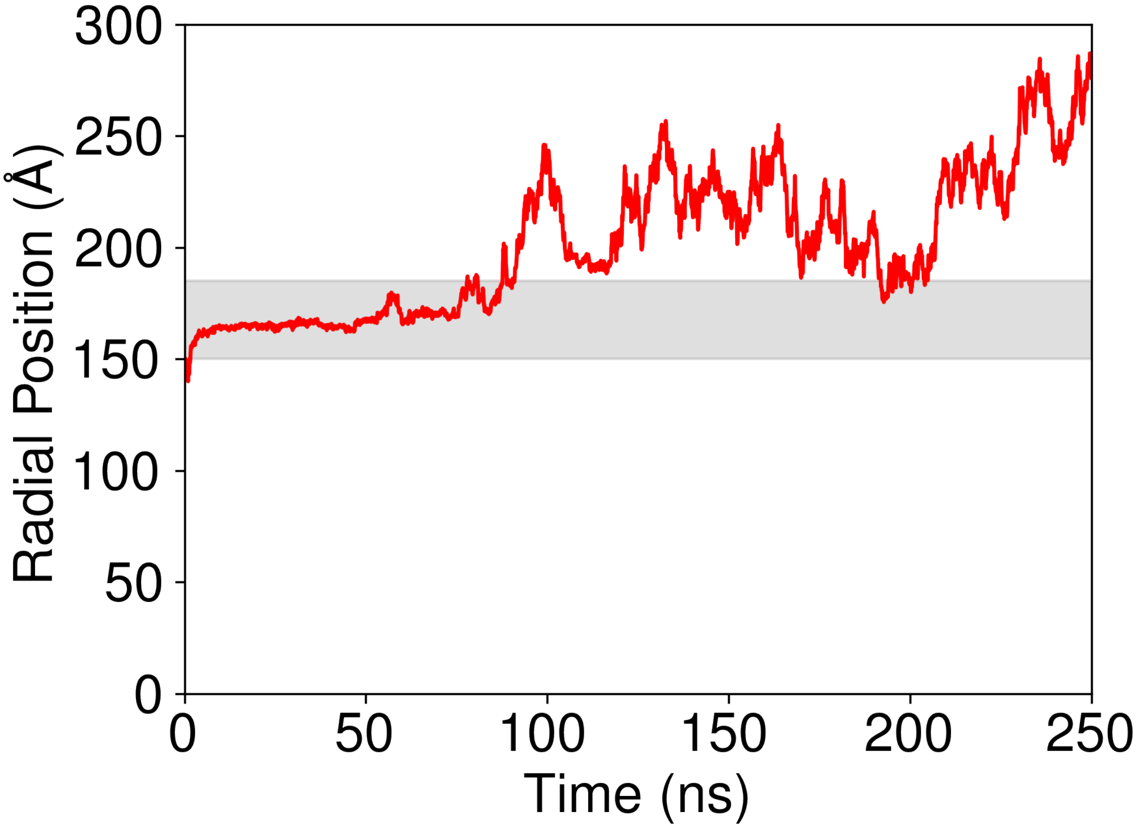

Figure S23: This is a trace for a G3P molecule crossing the BMC shell in simulation replica 2. The gray region indicates where the shell protein is.

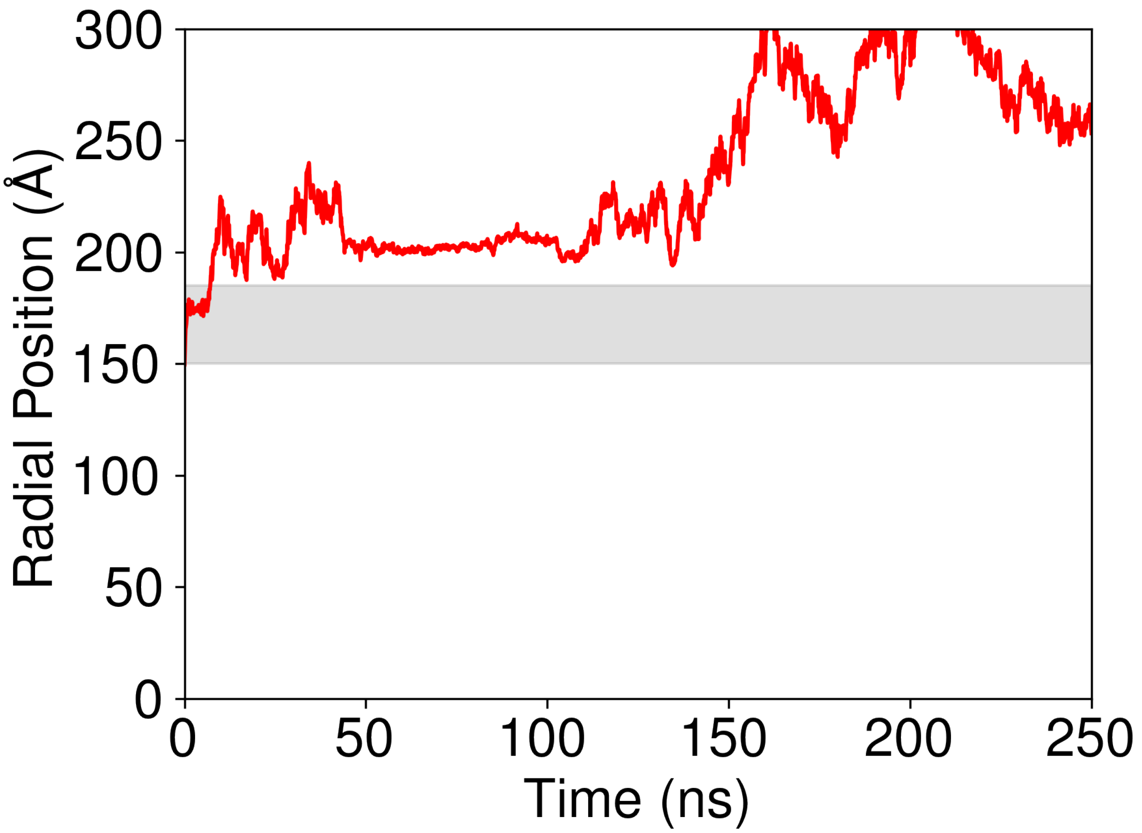

Figure S24: This is a trace for a G3P molecule crossing the BMC shell in simulation replica 3. The gray region indicates where the shell protein is.

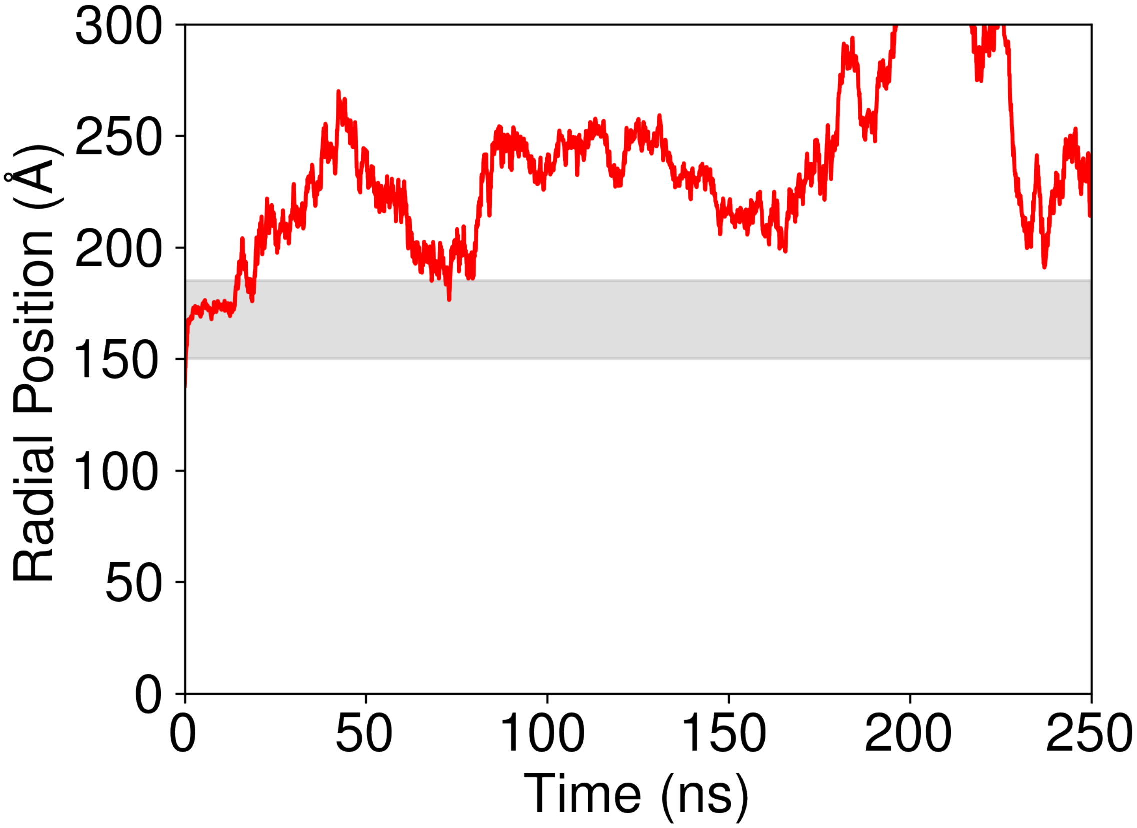

Figure S25: This is a trace for a G3P molecule crossing the BMC shell in simulation replica 3. The gray region indicates where the shell protein is.

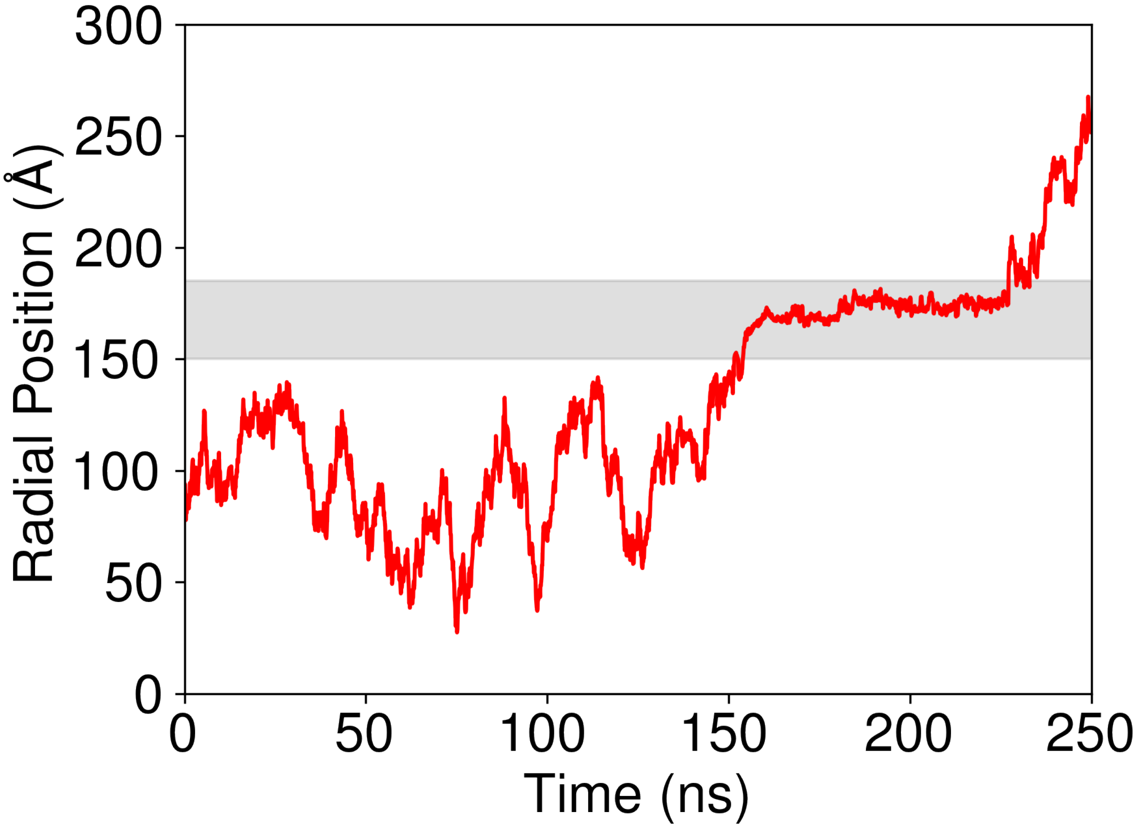

Figure S26: This is a trace for a G3P molecule crossing the BMC shell in simulation replica 3. The gray region indicates where the shell protein is.

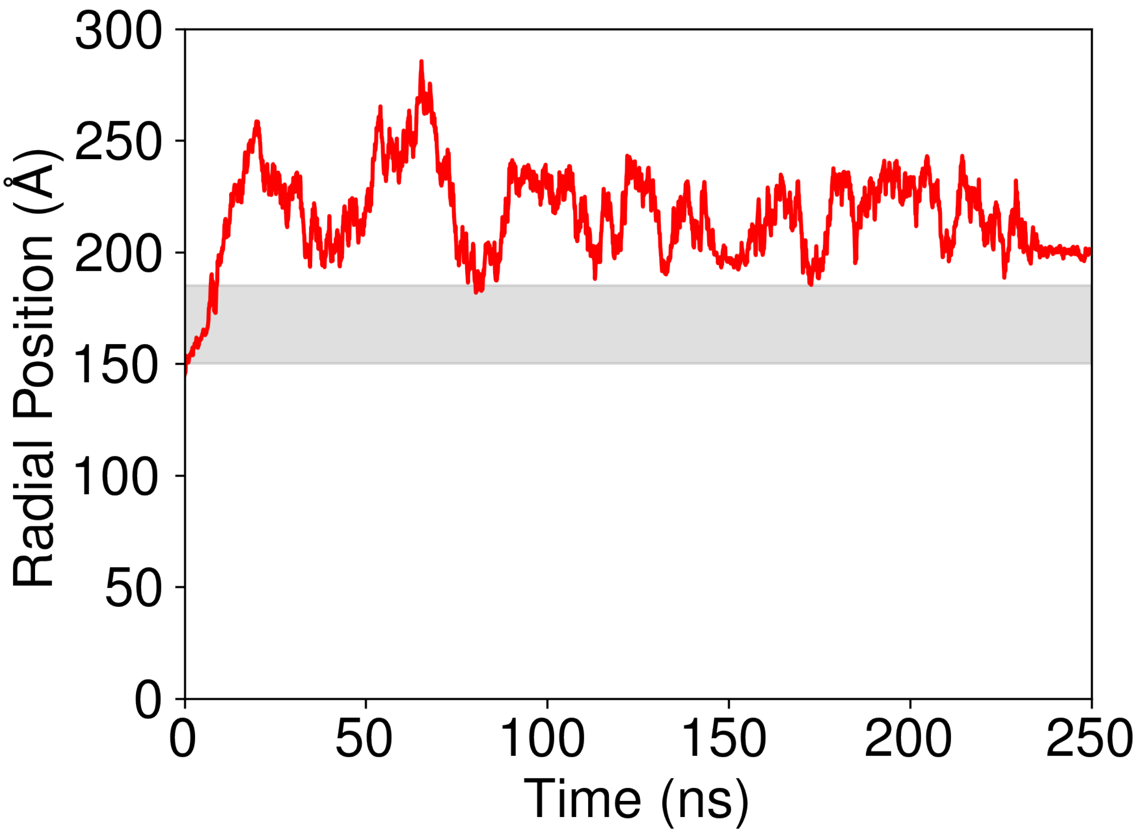

Figure S27: This is a trace for a G3P molecule crossing the BMC shell in simulation replica 3. The gray region indicates where the shell protein is.

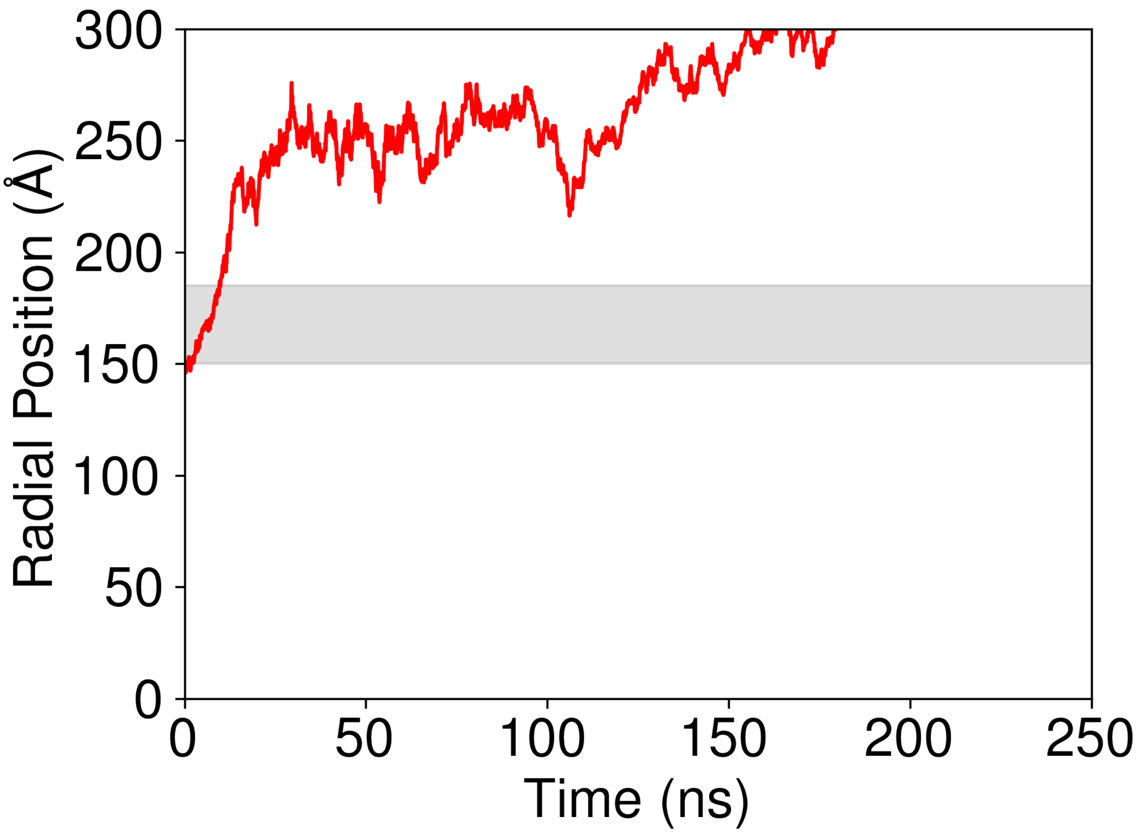

Figure S28: This is a trace for a G3P molecule crossing the BMC shell in simulation replica 3. The gray region indicates where the shell protein is.

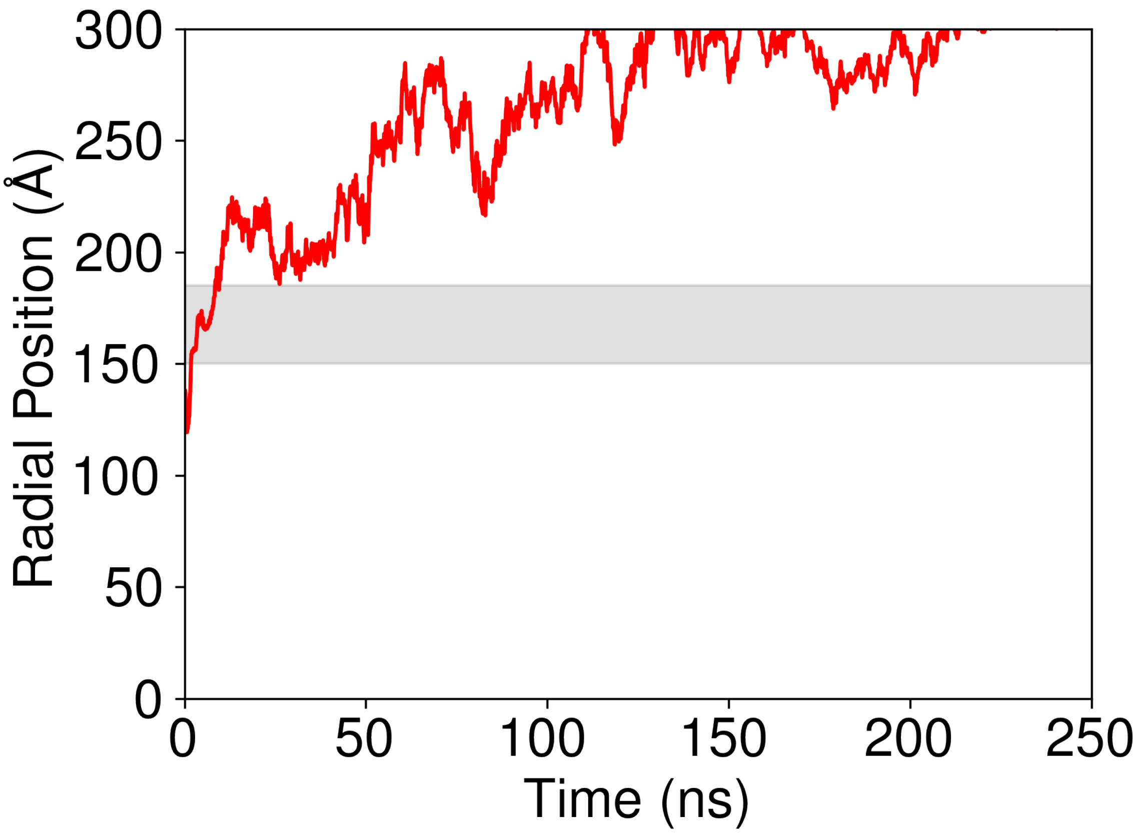

Figure S29: This is a trace for a G3P molecule crossing the BMC shell in simulation replica 3. The gray region indicates where the shell protein is.

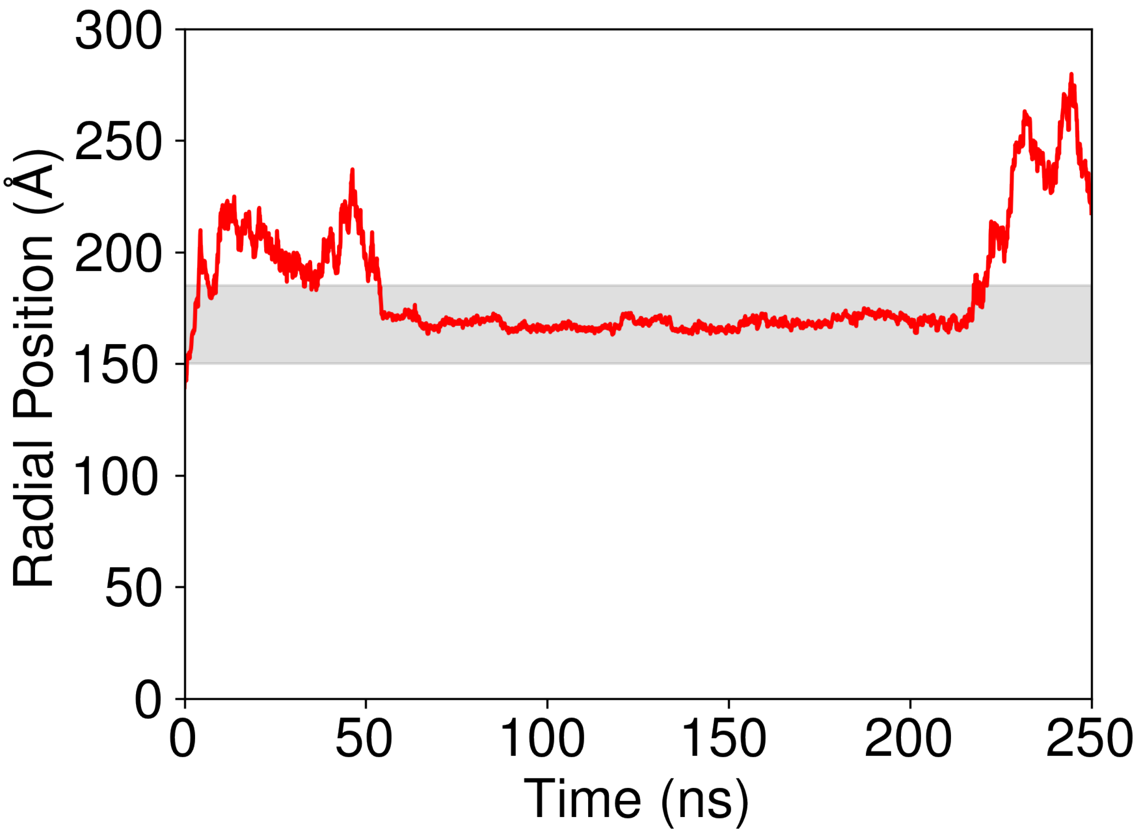

Figure S30: This is a trace for a G3P molecule crossing the BMC shell in simulation replica 3. The gray region indicates where the shell protein is.

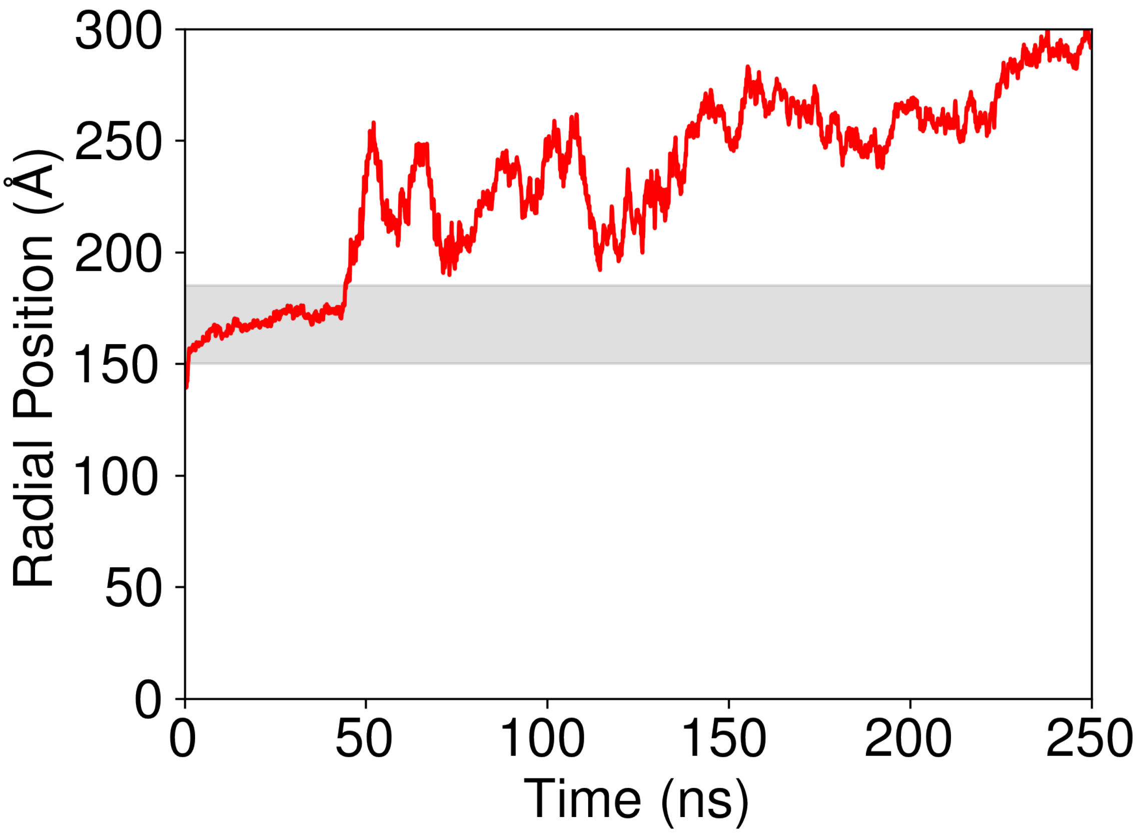

Figure S31: This is a trace for a G3P molecule crossing the BMC shell in simulation replica 3. The gray region indicates where the shell protein is.

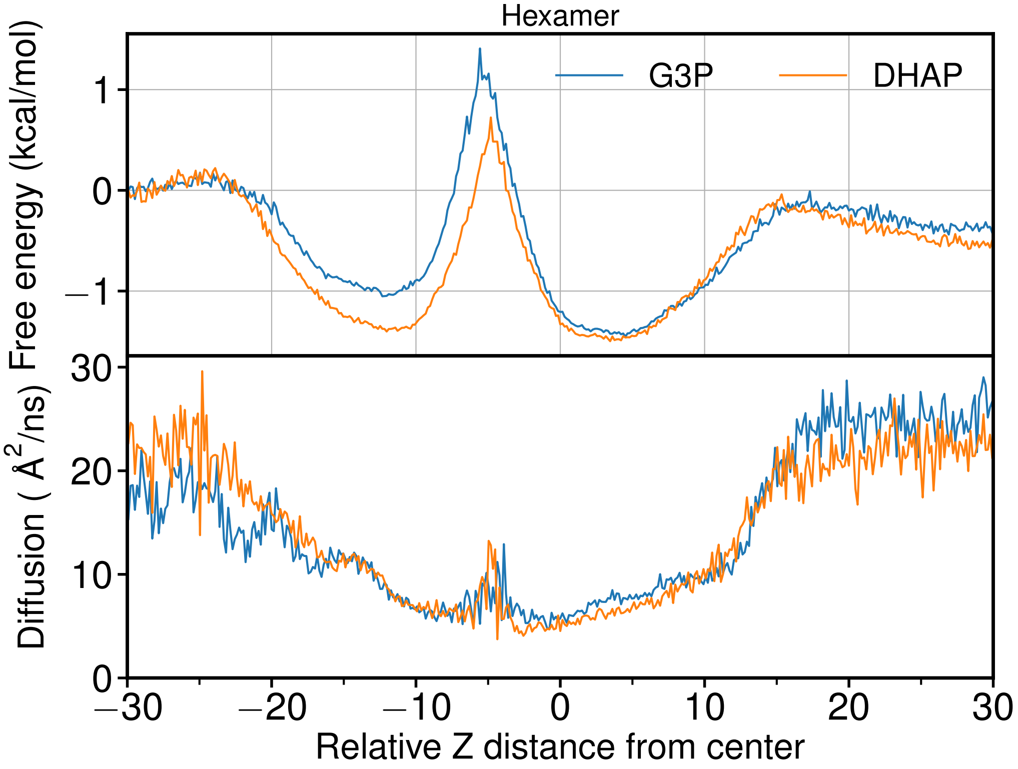

Figure S32: Diffusion and free energy plot of the DHAP and G3P molecules across the hexameric pore, obtained from the equilibrium trajectory.

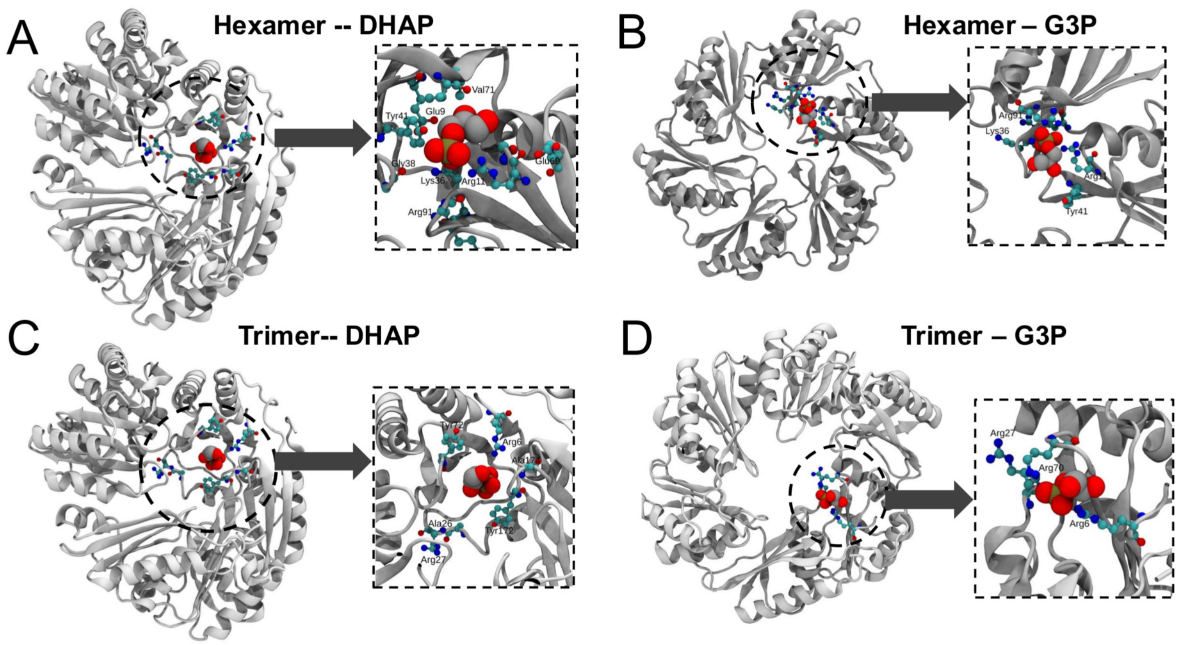

Figure S33: Interaction network formed between DHAP in the hexamer (A) and trimers (C). Panel C and D plots the interaction of G3P in hexamers (B) and trimers (D), respectively. The snapshot shows amino acids present within 5 Å of the metabolites. The quantification of the contacts can be found in Fig. S34. For clarity, only residues in the same z-plane as the metabolite are shown explicitly in the snapshots.

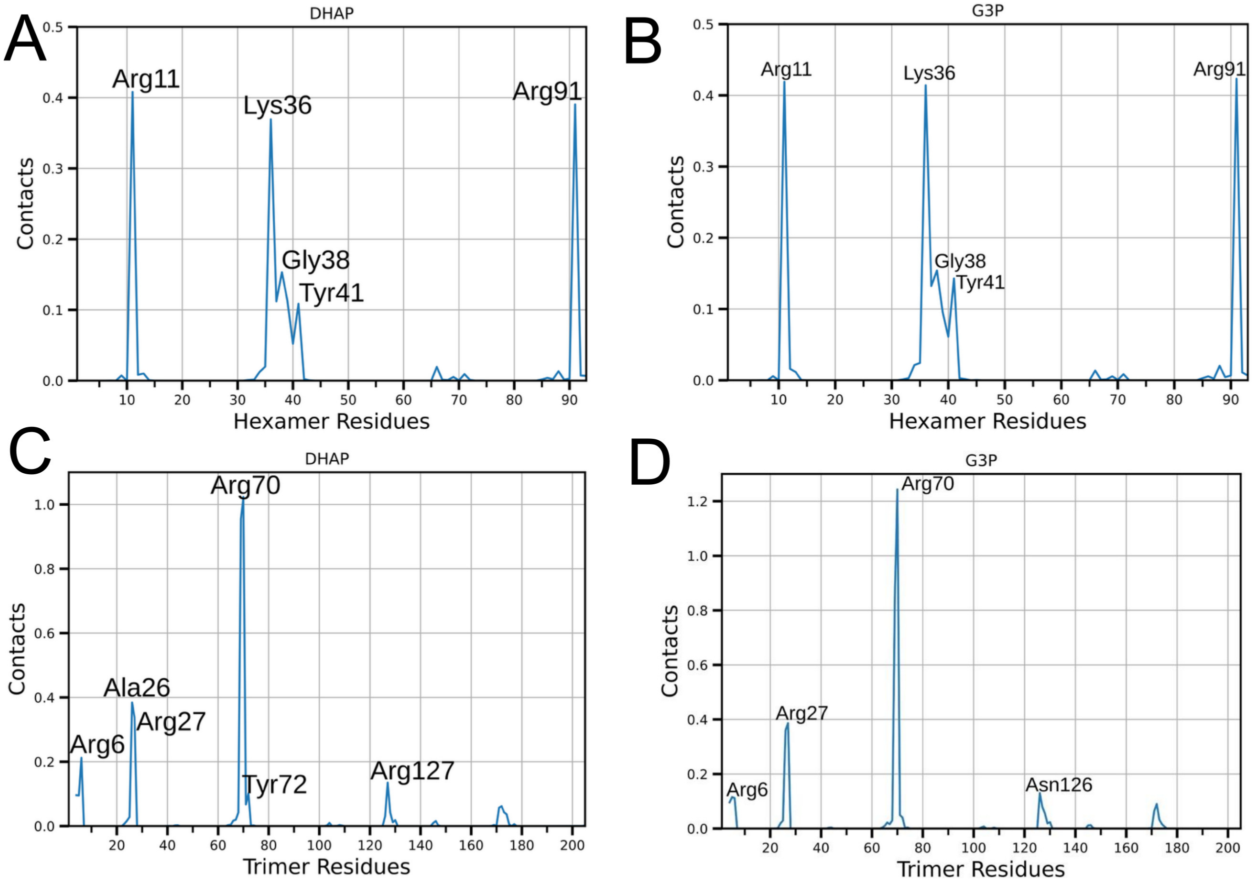

Figure S34: (A,C) Hexamer and (B,D) trimer residues interacting with DHAP (A,C) and G3P (B,D) within the REUS simulation trajectory. The measured contact numbers are determined via Eq. 1, and are accumulated by residue.

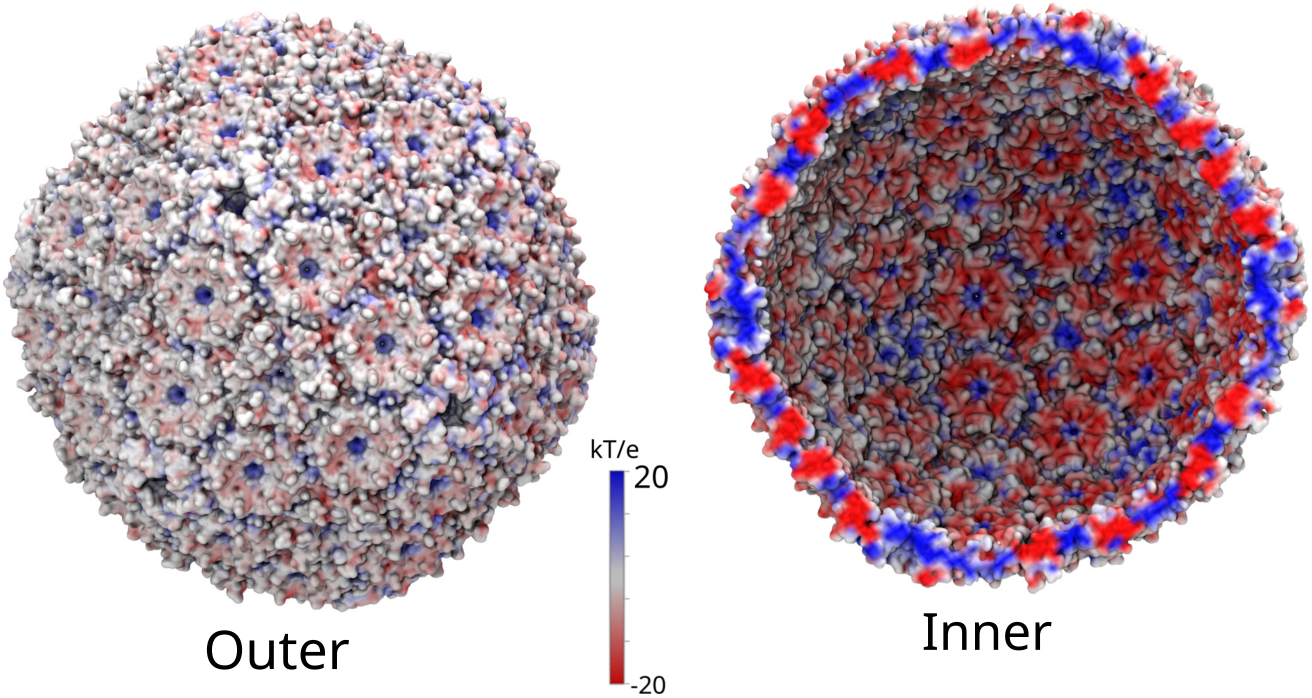

Figure S35: Electrostatic surface view of the outside and inside of BMC shell. Color gradient from -4 kT/e (red) to +4 kT/e (blue).

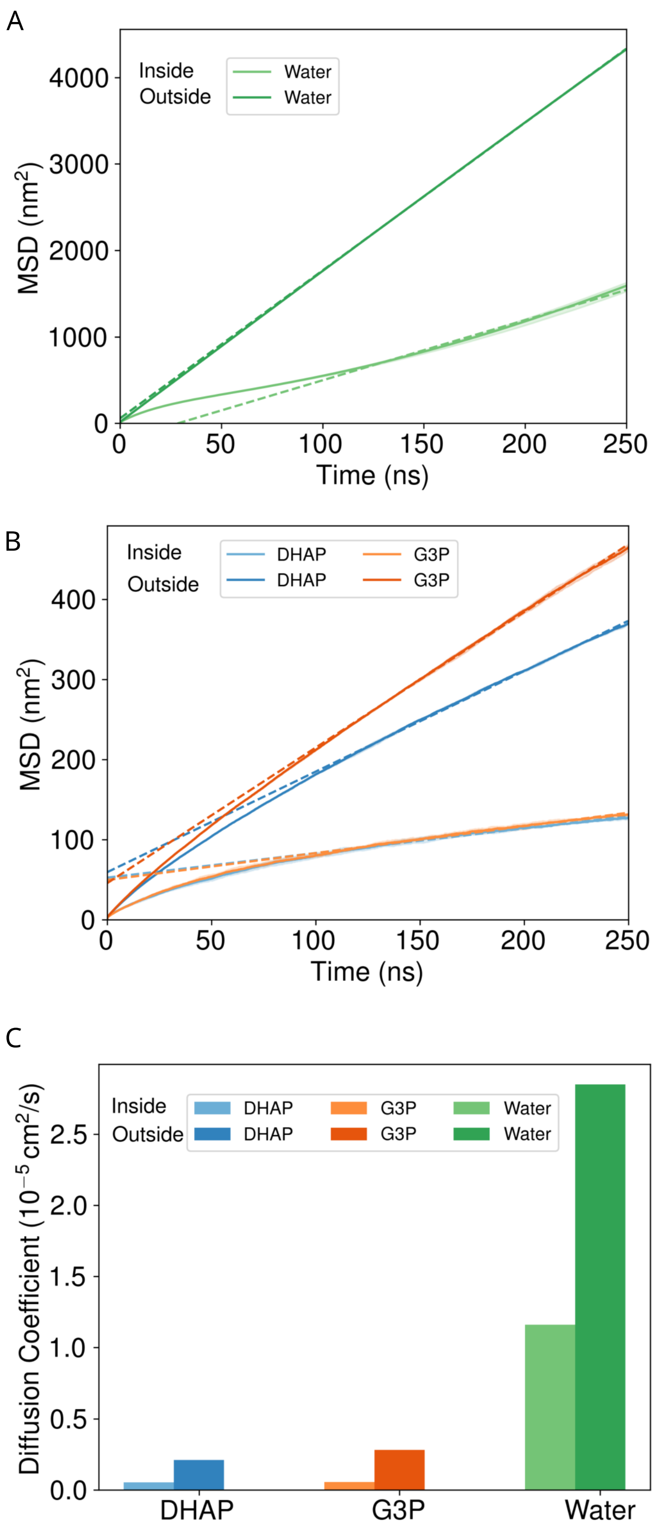

Figure S36: Mean Squared Displacement with respect to first frame of the simulation for water (A) and metabolites (B). Diffusion coefficient (C) calculated from equation 2 for DHAP (Blue), G3P(Red) and water (Green). The Darker color represent the MSD or diffusion coefficient outside the BMC and lighter color represent the inside rates.

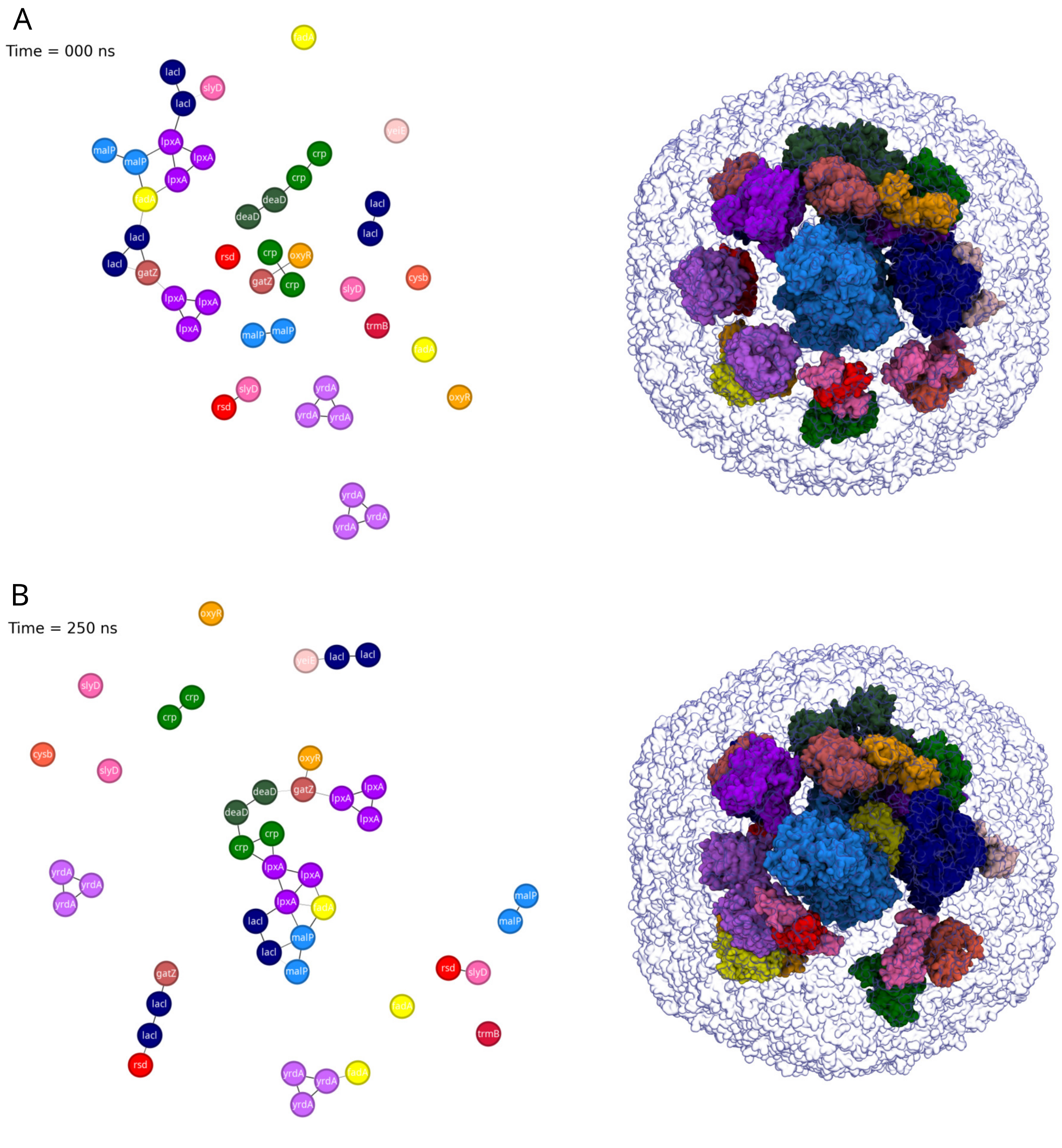

Figure S37: Cargo proteins intra interaction during the MD simulation (left) and snapshot of MD simualtion for the same time step (right) Snapshot. The first (A) and last (B) frame of the simulation along with interaction network for that frame. The protein are represented as surfaces, shell proteins are shown as transparent and cargo proteins as opaque chalky surface. Trimers are color coded in shades of purple, dimers are in the shades of blue and green and monomers are color coded in shades of red, yellow and orange.

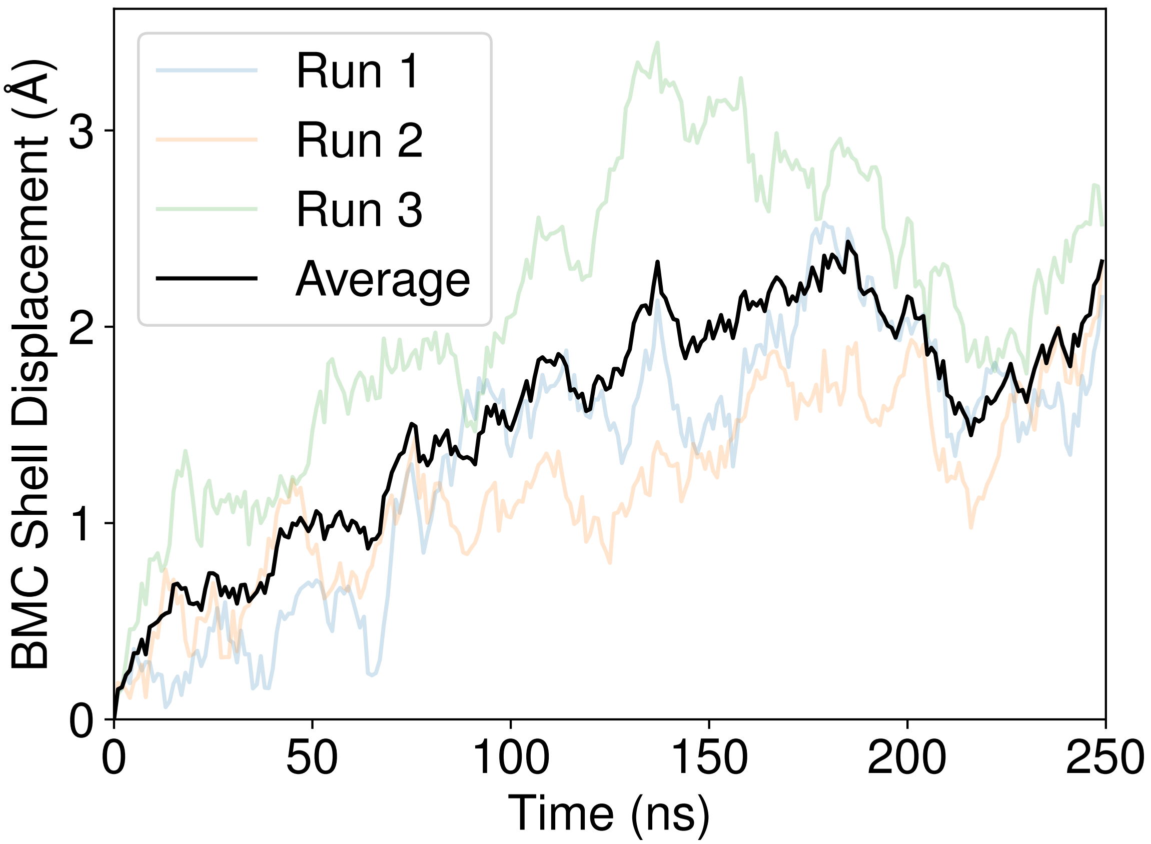

Figure S38: Quantifying the displacement of the center of mass for the HO shell proteins as a function of time. Due to the large size for the HO shell, the displacements are very small.

References

(S1) Zuo, X.; Jussupow, A.; Ponomarenko, N. S.; Grant, T. D.; Tefft, N. M.; Yadav, N. S.; Range, K. L.; Ralston, C. Y.; TerAvest, M. A.; Sutter, M.; Kerfeld, C. A.; Vermaas, J. V.; Feig, M.; Tiede, D. M. Structure Characterization of Bacterial Microcompartment Shells via X-ray Scattering and Coordinate Modeling: Evidence for Adventitious Capture of Cytoplasmic Proteins. *ACS Appl. Bio Mater.* **2025**, acsabm.4c01621.
